# Diffusion MRI: Assessment of the Impact of Acquisition and Preprocessing Methods Using the BrainVISA-Diffuse Toolbox

**DOI:** 10.3389/fnins.2019.00536

**Published:** 2019-06-07

**Authors:** Lucile Brun, Alexandre Pron, Julien Sein, Christine Deruelle, Olivier Coulon

**Affiliations:** Institut de Neurosciences de La Timone, Aix-Marseille University, CNRS, UMR 7289, Marseille, France

**Keywords:** brain, DWI, distortions, preprocessing, toolboxc

## Abstract

Diffusion MR images are prone to severe geometric distortions induced by head movement, eddy-current and inhomogeneity of magnetic susceptibility. Various correction methods have been proposed that depend on the choice of the acquisition settings and potentially provide highly different data quality. However, the impact of this choice has not been evaluated in terms of the ratio between scan time and preprocessed data quality. This study aims at investigating the impact of six well-known preprocessing methods, each associated to specific acquisition settings, on the outcome of diffusion analyses. For this purpose, we developed a comprehensive toolbox called *Diffuse* which automatically guides the user to the best preprocessing pipeline according to the input data. Using MR images of 20 subjects from the HCP dataset, we compared the six pre-processing pipelines regarding the following criteria: the ability to recover brain’s true geometry, the tensor model estimation and derived indices in the white matter, and finally the spatial dispersion of six well known connectivity pathways. As expected the pipeline associated to the longer acquisition fully repeated with reversed phase-encoding (RPE) yielded the higher data quality and was used as a reference to evaluate the other pipelines. In this way, we highlighted several significant aspects of other pre-processing pipelines. Our results first established that eddy-current correction improves the tensor-fitting performance with a localized impact especially in the corpus callosum. Concerning susceptibility distortions, we showed that the use of a field map is not sufficient and involves additional smoothing, yielding to an artificial decrease of tensor-fitting error. Of most importance, our findings demonstrate that, for an equivalent scan time, the acquisition of a b0 volume with RPE ensures a better brain’s geometry reconstruction and local improvement of tensor quality, without any smoothing of the image. This was found to be the best scan time/data quality compromise. To conclude, this study highlights and attempts to quantify the strong dependence of diffusion metrics on acquisition settings and preprocessing methods.

## Introduction

Diffusion-weighted imaging (DWI) has established itself as a reference technique for the *in vivo* inference of structural brain connectivity and for the investigation of white matter microstructure (Hagmann et al., 2010; Ghosh and Deriche, 2016). If echo-planar imaging (EPI) sequences, commonly used in DWI, provide a high signal to noise ratio (SNR) and rapid scan time, they are nonetheless prone to severe artifacts such as non-zero off-resonance fields (Le Bihan et al., 2006) stemming from the discontinuity of magnetic susceptibility of the tissues and from eddy-currents induced in the nearby conductors. The low bandwidth in the phase-encode direction makes EPI sequences particularly sensitive to these two artifacts which disrupt the spatial encoding gradients (Schmitt et al., 1998). These artifacts can thus induce important geometric distortions due to a voxel-shift in the signal reconstruction, which may lead to wrong interpretations if not corrected properly (Embleton et al., 2010; Yendiki et al., 2014). Despite important technical advances to achieve high quality diffusion signal modeling and fibers reconstruction (e.g., Auría et al., 2015; Ning et al., 2015; Girard et al., 2017; Bastiani et al., 2019), the quality of the preprocessing is should not be neglected (Jones and Cercignani, 2010). Yet, last advances in this area have mostly concerned research-type acquisition protocols (Sotiropoulos et al., 2013; Glasser et al., 2016; Bastiani et al., 2017). Transfer from research to clinical context is still limited because of the complexity of correction methods.

Magnetic susceptibility differences between tissue, air and bone alter the B0 magnetic field and result in local MR frequency variations at tissue interfaces such as the sphenoid sinus, temporal lobe and brain stem. Such susceptibility-induced gradients interfere with the spatial encoding gradients and may cause signal dropout and geometric distortions. Susceptibility-induced distortions do not depend on diffusion gradients and remain constant across volumes, assuming that head movements are not excessive. The susceptibility-induced distortions can be corrected either by measuring the magnetic field at the acquisition using an additional sequence or by estimating it *a posteriori*. The former approach – field map-based – consists in an additional double-echo acquisition (Jezzard and Balaban, 1995; Reber et al., 1998) where the phase difference between the two echoes is used to estimate a B0 field map. This field map is used to estimate the non-linear voxel-wise shift and the signal loss. This method, however, suffers from the non-linearity of susceptibility-induced distortions that causes neighboring voxels to collapse into a single one resulting in an ill-posed problem of intensity retrieval and a potential loss of information (Jones and Cercignani, 2010). In the latter approach – image-based – the distorted magnetic field can be estimated in two ways. One way consists in computing the non-linear deformation field between diffusion-weighted and anatomical (T1 or T2) images, which suffers the same issues as the field map-based approach. The other way consists in acquiring additional non-diffusion weighted volumes with reversed phase-encode direction (FSb0RPE) (Andersson et al., 2003). In this way, the complementary information contained by opposed FSb0RPE images allow recovering the full intensity information.

Diffusion-weighted imaging is also affected by eddy current artifacts due to the rapid switch of strong diffusion encoding gradients which generates electric currents in the nearby conductors, inducing local magnetic fields that interfere with the spatial encoding gradient (Jezzard et al., 1998). Eddy-current artifacts typically induce shearing, stretching and/or compression along the phase-encode direction which add up to the motion-induced translations and rotations and lead to a misalignment between successive volumes. Unlike magnetic susceptibility induced distortions, these effects vary across diffusion gradient orientations and are enhanced by the fact that higher b-values require the application of stronger diffusion gradients for longer periods. To a first approximation, eddy-currents can be considered as originating from a linear combination of the linear gradient coil fields. Hence, a simple affine transformation can be applied to correct for eddy-current induced distortions as well as head movements (Haselgrove and Moore, 1996). This method does not require any specific acquisition but is less appropriate for high *b*-value associated to signal attenuation and increased contrast variation between images (Ben-Amitay et al., 2012) where the affine registration fails to correct the eddy-currents completely. Furthermore, the linear assumption is no longer verified for modern scanners where stronger gradients have a high degree of non-linearity (Haselgrove and Moore, 1996). Indeed, authors in Rohde et al. (2004) and Andersson and Sotiropoulos (2016) have shown that higher order models provided a better fit of the off-resonance field caused by eddy-currents. To correct properly reconstruct the signal intensity, such techniques exploit the fact that two images acquired with reversed diffusion gradient directions or reversed phase-encoding (RPE) directions would have similar diffusion contrast but reversed eddy-current distortions (e.g., Bodammer et al., 2004; Shen et al., 2004; Embleton et al., 2010). These reversed gradient methods require at least that gradients are sampled over the full sphere or that each diffusion gradient is repeated twice with reversed polarity.

This interdependence between a variety of acquisition settings and a variety of correction methods can result in very different diffusion metric assessments and connectivity inferences. Therefore, the choice of acquisition settings has obviously a crucial role in the interpretation of results. This choice – usually driven by the time constraint typically different between clinical and research contexts – should also be done in the light of analyses comparing its influence on final diffusion measurements. For instance, for an equivalent time cost, it is yet debatable whether the acquisition of a B0 field map image would conduct to a better data quality than the acquisition of a single b0 volume with reversed phase encoding direction. Conversely, in a context of uncontrolled external acquisition such as for public or multicentric datasets, the limitations inherent to the quality of the associated pre-treatments are still poorly documented. To date, quantitative comparisons of preprocessing techniques have only been performed in a clinical (e.g., Cusack et al., 2003; Wang et al., 2017) or a research context (e.g., Rohde et al., 2004). A comprehensive evaluation of the existing and widely used pre-processing methods and their dependence on acquisition settings is still needed.

In the current study, we adopt a holistic approach, to quantify the influence of various acquisition settings and the entire associated preprocessing pipeline on typical DWI measurements, including diffusion and tractography quantification. A holistic approach is crucial for two reasons. First, for a given configuration of acquisition settings, it should help orienting the interpretation of results as well as any inter-study comparison according to the limitations imposed by the corresponding preprocessing methods. Second, for a given scope of analysis, it should guide the choice of acquisition settings in the light of the best data quality for acquisition time constrain. For this purpose, we developed a dedicated and comprehensive toolbox called *Diffuse* which, out of six different preprocessing pipelines, automatically selects the one most adapted to the acquisition settings. *Diffuse* also includes registration methods as well as post-processing methods to perform local signal modeling and tractography. Six different types of acquisition settings were chosen because they are widely used in the literature ranging from clinical to research contexts, and each of them matches to a known dedicated preprocessing pipeline. We selected MRI data of 20 subjects from the HCP database (Van Essen et al., 2013) which is, to our knowledge, the only database that includes all the data necessary to conduct this work. To relate the same experiments in a clinical context, we also acquired a similar acquisition set for one healthy subject in a standard 3T scanner and with lower spatial resolution. In the next section, we describe the six subsets extracted from these data as well as the preprocessing pipelines involved. Then, an overall comparison of the distortion correction pipelines is performed through four different experiments. First, we quantified their ability to recover brain geometry, which is an important step in the normalization process for group measurements, using a similarity measure between the corrected DWI and T1w images. Second, both qualitative and quantitative metrics were used to assess the influence of preprocessing pipelines on the quality of diffusion tensor estimation in white-matter tissues. The same experiment was then performed at a local scale to evaluate the impact on central white-matter regions that are of major interest for pathology studies and used as seeds for tractography. Finally, we used a quantitative metric of tract spatial dispersion to evaluated the distal impact of preprocessing pipelines on the reconstruction of six well-known bundles.

## Preprocessing Pipelines

### Data

#### HCP Dataset

The HCP dataset (Van Essen et al., 2013) was found as the only publicly available database including every MRI sequences and acquisition settings necessary to pre-process images through the six pipelines. MRI data of 20 participants were used in this study (subject IDs are listed in the Section “Annexe”). All individuals were right-handed males (age range 25–30). Images were acquired using a modified version of Siemens Skyra 3T scanner (Siemens, Erlangen, Germany) with a maximum gradient strength of 100 mT/m, slew rate of 200 T/m/s and a 32-channel head coil. T1-weighted images were acquired using 3D MPRAGE sequence (TR/TE = 2400/2.14 ms, flip angle = 8°, FOV = 224 × 224 mm^2^, resolution = 0.7 mm isotropic). Diffusion-weighted images were acquired with a spin-echo EPI sequence consisting of 3 shells of 90 diffusion-weighted volumes each (*b* = 1000, 2000, and 3000 s/mm^2^) and 6 interleaved b0 volumes each (TR/TE = 5520/89.5 ms, resolution: 1.25 mm isotropic, FOV = 210 × 180 mm^2^, 111 axial slices, multiband factor = 3, partial Fourier = 6/8, echo spacing = 0.78 ms). Gradients directions were sampled over the entire sphere, using the electrostatic repulsion method (Caruyer et al., 2013). The entire diffusion sequence was repeated twice with RPE (L- > R, R- > L). A B0 field map image was also acquired using a dual-echo gradient-echo sequence (with delta TE = 2.46 ms, resolution: 2 mm isotropic). Note that the DW images were acquired during a different session from the T1 and field map images. Different subsets of these data were extracted in order to be compatible with the requirements of the six preprocessing pipelines, as detailed in Table 1. In particular, to be comparable, the 6 subsets share the same basis consisting in 3 shells of 90 gradient directions and 6 b0 volumes.

**Table 1:** Acquisition data subsets extracted for each of the six preprocessing pipelines.

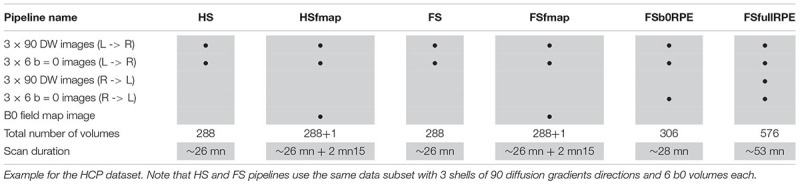

#### Clinical Dataset

The MRI images of a healthy volunteer were acquired using the same sequences with clinical settings. Results from this clinical dataset have no statistical value and are shown to illustrate the consistency of the results even with data other than high quality HCP scans. In particular, we used this data to illustrate the impact of *b*-value and spatial resolution on the same analyses. A thorough description of the acquisition settings and results can be found in Supplementary Material S1.

### Data Processing: The *Diffuse* Toolbox

*Diffuse* is a BrainVISA toolbox, written in the Python language dedicated to diffusion MRI processing and publicly available on Github^1^. *Diffuse* relies on algorithms from FSL^2^ (Jenkinson et al., 2012), Dipy^3^. (Garyfallidis et al., 2014), Niftyreg^4^. (Modat et al., 2010) and on functionalities provided by the BrainVISA software platform^5^ for neuroimaging (Geffroy et al., 2011). This platform already offers several processing pipelines for other modalities such as structural and functional MRI. In particular, an anatomical pipeline gives access to segmented T1w images, cortical surface meshes and a number of tools providing morphometric and functional measurements on the surface. BrainVISA includes the Anatomist software (Rivière et al., 2011) for visualization and interaction with all associated data formats. All processes can be operated under a unified graphical user interface or as batch and using parallel distribution for processing groups of subjects.

**FIGURE 1:**
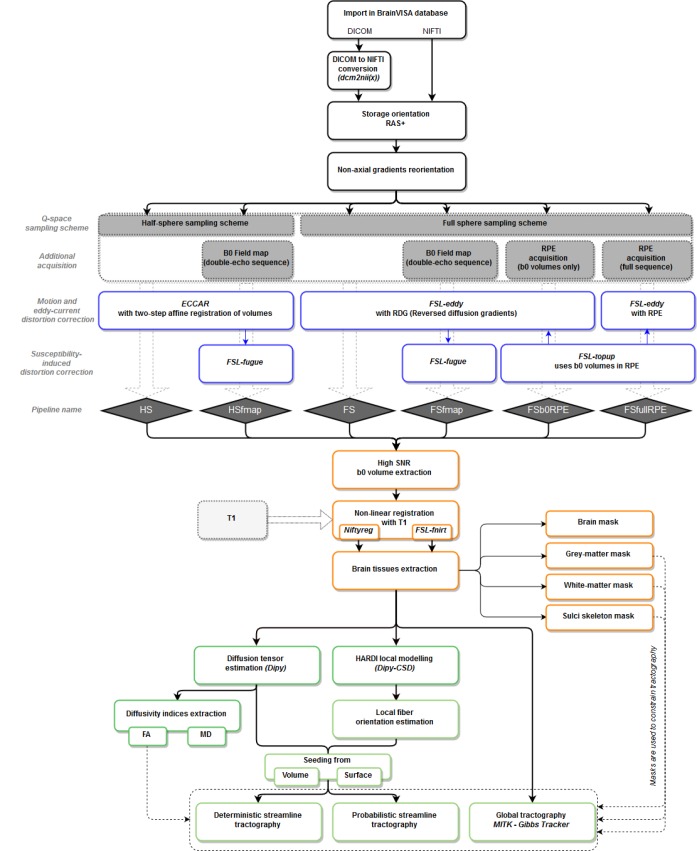
Workflow of the *Diffuse* toolbox for DWI data processing implemented in the *BrainVISA* software platform. It includes the four parts detailed in the Section “Data Processing: The *Diffuse* Toolbox” of the manuscript. (In black) Importation, conversion and reorientation of data into the *BrainVISA* database. (In blue) Distortion correction through the six pre-processing pipelines derived from different acquisition settings. Most of the correction methods use *FSL* tools. The pipelines’ names are indicated in blue diamonds. (In orange) The registration with the structural space is performed with either *Niftyreg* or *FSL*. Anatomical masks are extracted from the registered T1 data and can be used to constrain tractography. (In green) The data post-processing includes diffusion model estimation (tensor or CSD) and tractography (deterministic, probabilistic or global). All processes use *Dipy* tools except the global Gibbs tracking package implemented in Matlab.

T1w MR images were processed using BrainVISA’s Morphologist pipeline, dedicated to the processing of anatomical images (Fischer et al., 2012), to obtain bias corrected T1w images as well as brain extraction, gray and white matter masks and cortical surface meshes. Diffusion-weighted images were processed through the Diffuse workflow described in Figure 1. It consists in four steps that are detailed below: (1) importation and reorientation of data, (2) distortion corrections, (3) structural to diffusion space registration and (4) diffusion model estimation and tractography.

#### Importation and Reorientation of Data

Data files are stored into a database to facilitate filesystem organization and indexation. This is an important practical aspect in the management of diffusion data with various complex data types and, in our case, for testing multiple processing using varying parameters. While input files are imported into the BrainVISA database, the storage orientation of DWI data and gradients vectors are changed to the neurological convention (RAS+)^6^ which is supported by both FSL and Dipy tools.

#### Distortion Corrections

##### Motion and eddy-currents induced distortions

Motion and eddy-current-induced distortions were corrected using three different methods.

The first method consists in using an affine registration, considering the distortions as a linear combination of translation, rotation, scaling and shearing. In *Diffuse*, we implemented a method called ECCAR (Eddy-Currents Correction by Affine Registration), derived from the previous ‘*eddy_correct’* tool of FSL (version 5.0.9 and anterior), to align all diffusion-weighted images to the first non-diffusion weighted volume using a two-step approach. To ensure minimal error due to intensity differences between b0 and T1w images (Rohde et al., 2004; Ben-Amitay et al., 2012), volumes are first aligned to the closest interspersed b0 volumes, which are in turn aligned to the first one. For the same reason, we used the mutual information cost function which is adapted to multimodal registration. The two transformations are combined to apply a single resampling to each volume, with a spline interpolation. This single correction step does not require any additional acquisition and constitutes the first preprocessing pipeline called hereafter “**HS pipeline**” (Half-Sphere), in contrast to the full-sphere sampling condition required for the following methods. Note, however, that in this article, for comparison purpose, we applied this pipeline to the first subset of DWI data containing 90 multi-shell diffusion gradient directions sampled over the full sphere and 6 b0 volumes with LR phase-encoding direction.

The second method uses the fact that gradients directions have been sampled over the full sphere. With a sufficient number of samples, images with quasi-opposed gradients directions can be considered with opposed distortions. This method implemented in *Diffuse* calls the ‘*eddy’* tool from the FSL software (Andersson and Sotiropoulos, 2016). Using pairs of volumes with close orientation but quasi-opposed polarity of diffusion gradients, the algorithm applies a non-parametric Gaussian Process to estimate a higher order distortion field caused by both eddy-currents and motion and recover the midway geometry in the image. During the final resampling, a spline interpolation is combined with a Jacobian modulation to account for signal dilution in areas with stretching. This single correction step constitutes the preprocessing pipeline called hereafter “**FS pipeline**” (Full-Sphere) and could be applied to the same first subset. The method is also embedded in two other pipelines, depending on the magnetic susceptibility-induced distortion method that is used in conjunction, as described in the next sub-section.

The third method also calls the *‘eddy’* tool from FSL, but with a different resampling technique. The repetition of all diffusion gradient directions using the RPE direction RL provides a mean to resolve signal intensity recovery in compressed areas where signal has piled-up, with a least-squares reconstruction (Andersson and Sotiropoulos, 2016). This method leads to the pipeline called hereafter “**FSfullRPE.**” It should be emphasized, however, that this method requires twice as much acquisition time as compared to the two methods described above.

Note that in the three methods, to preserve the directional information of DWI data, the diffusion gradient vectors are reoriented using the same rotation parameters used to transform each volume during motion and eddy-current correction. This step is critical to correctly estimate the diffusion parameters and fiber orientation (Leemans and Jones, 2009; Jones and Cercignani, 2010).

##### B0 susceptibility-induced distortions

In *Diffuse*, two approaches were implemented to correct for magnetic susceptibility-induced distortions.

The first procedure uses the acquired B0 field map magnitude and phase images to correct the data through the workflow described in Cusack et al. (2003) involving the *‘fugue’* command from FSL (Jenkinson et al., 2012). This correction step is applied after eddy-current and motion correction to ensure that volumes, and thus head-dependent distortion fields, are aligned. This yield two other preprocessing pipelines called **“HSfmap pipeline”** and **“FSfmap pipeline.”**

The second method uses non-diffusion weighted volumes acquired with reversed phase-encode direction (FSb0RPE) (Andersson et al., 2003). In the *Diffuse* toolbox, this approach is implemented via the use of the *‘topup’* tool from FSL (Smith et al., 2004). ‘*Topup’* combines pairs of b0 images with opposed distortions to estimate the susceptibility-induced off-resonance field. This distortion field is used as input in the *‘eddy’* tool which correct simultaneously for susceptibility, eddy-current distortions and movements. A subset of DWI data containing 90 multi-shell diffusion gradient directions and 6 b0 volumes with LR phase-encoding direction plus 6 b0 volumes with RL phase-encoding direction was processed through the “**FSb0RPE pipeline.”** The full subset with 90 multi-shell diffusion gradient directions repeated in both LR and RL phase-encoding directions was processed through the **“FSfullRPE pipeline.”**

#### Structural to Diffusion Space Registration

After distortion correction, all non-diffusion weighted volumes are averaged to create a high SNR b0 image registered into the T1w image referential using non-linear registration. Two methods have been integrated in the toolbox, using either *‘fnirt’* from FSL (Andersson et al., 2009) or *‘reg f3d’* from Niftyreg^7^ (Modat et al., 2010) (Figure 1). For both methods, an initialization step is done using the rigid body transformation of *‘flirt’* from FSL. Note that the transformation is first estimated between the fractional anisotropy map and the T1w image which show similar gray-white contrasts and then applied to the b0 image. For our experiments, we use *‘reg f3d’* which outperformed *‘fnirt’*. In particular, ‘*fnirt’* failed to align regions with high intensities in the FA map such as the brain stem and the corpus callosum (data not presented in this article).

#### Diffusion Model Estimation and Tractography

Two diffusion models and three tractography algorithms constitute the post-processing steps implemented in the toolbox (Figure 1). For our experiments, the diffusion tensor was estimated using Dipy (Garyfallidis et al., 2014) from which were extracted tensor-derived indices such as eigenvalues, eigenvectors, FA and MD [equations (4) and (5) in Section Experiment 2], as well as the signal prediction and the tensor fitting error [TFE, equation (2)]. To perform tracts reconstruction, we used the global Gibbs tracker proposed by Reisert et al. (2011) which consists in estimating fibers trajectory simultaneously in all voxels of the brain in a reasonable computational time. Global tractography does not require any seeding strategy and is more robust to local errors in the fiber orientation estimation than deterministic and probabilistic tractography algorithms (Reisert et al., 2011; Mangin et al., 2013).

## Experiments and Results

In this section, we investigated the performance of the six preprocessing pipelines on the HCP data in four different experiments, regarding: (1) their capacity to recover brain geometry, (2) their influence on whole-brain diffusivity measurements; (3) their influence on diffusivity in central white-matter regions; (4) their influence on tractography measurements. Experiments 1 and 2 were reproduced on the clinical data, for the multi-shell subset as well as for the 3 separated *b*-values. Note that this dataset was not included in the statistical analyses. For each experiment, complementary analysis was also performed to compare the data corrected through the six preprocessing pipelines with the raw uncorrected data. Results can be found in Supplementary Materials and interpretations will be drawn in the “Discussion” Section.

### Experiment 1: Performance of Distortion Correction Methods to Recover Brain Geometry

The performance of each distortion correction pipeline was assessed by measuring the similarity between the DWI and the T1w images as done in Cusack et al. (2003). Indeed, after correction for EPI distortions the brain should recover its initial geometry and the similarity with the T1w (considered as non-distorted) image should increase. To preserve local geometry, we only used the initialization step described in Section “Structural to Diffusion Space Registration” to rigidly align the average b0 image onto the T1w. Then, we computed the MMI as a similarity metric between the two images (Mattes et al., 2001). The main effect of distortion correction was evaluated using a one-way repeated measure ANOVA (RM-ANOVA). Reported effect sizes correspond to partial eta-squared (η_p_^2^) of the within-subject design, defined as follows:

$${\text{η}}_{\text{p}}^{2}=\frac{SS_{\text{effect}}}{SS_{\text{effect}}+SS_{\text{error}}}\;\;\;\;\;\;\;\;\;\;\;\;\;\;\;\;\;\left(1\right)$$

with *SS_effect_* the sum of squares of the effect and *SS_error_* the sum of squares of the error associated with the effect.

Then, the differences between pipelines of distortion correction were assessed using Student’s paired samples *t*-tests. The significance threshold was set to 0.003 (0.05/15pairs) to account for multiple comparisons.

**FIGURE 2:**
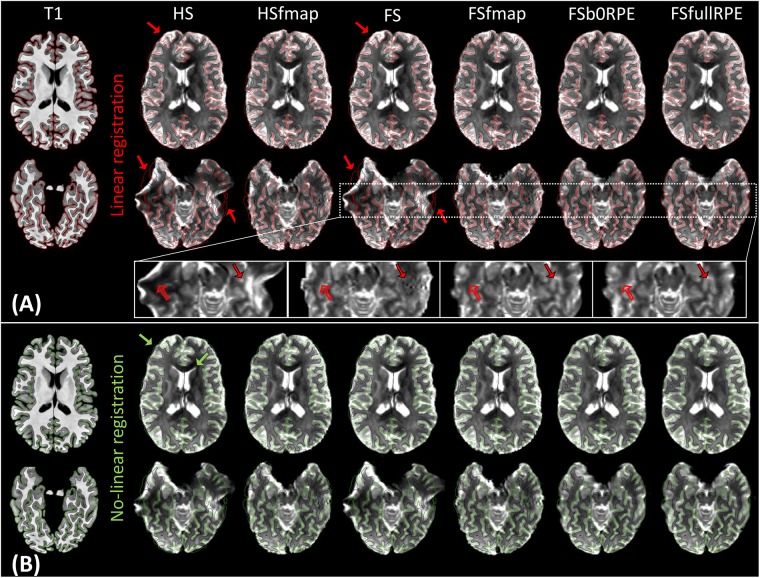
Average b0 image of one subject linearly **(A)** and non-linearly **(B)** registered into the structural space, after distortion correction through the six pipelines. Gray-white interface (black line) and cortical surface (red/green line) of the non-distorted T1w image are overlaid on the b0 image. **(A)** Susceptibility-induced distortions correction enables to recover the true geometry of the brain (red arrows). The signal intensity in stretched areas can be corrected using a B0 field map image (see empty arrows in the zoomed images). But only the use of a reversed phase-encoding acquisition (FSb0RPE and FSfullRPE) can properly reconstruct the signal in compressed areas (see full arrows). Particularly one can observe that the ringing artifact coming from the correction with fmap is not visible after the correction with topup (FSb0RPE and FSfullRPE). **(B)** Non-linear transformation is able to partially correct for residual geometric distortions in particular with a proper geometry of the frontal, temporal lobes and ventricles (green arrows).

Figure 2A illustrates the results of linear registration of the average b0 image onto the T1w image, for one subject (see Supplementary Material S2 for the results on clinical dataset). Our results show that the brain geometry in the frontal and temporal lobes (red arrows) are recovered only after explicit correction for susceptibility-induced distortions (HSfmap, FSfmap, FSb0RPE, and FSfullRPE pipelines). For data processed through the HS and FS pipelines, where susceptibility-induced distortions are not corrected explicitly, images contain high-intensity regions resulting from signal pile-up from surrounding voxels (full arrows) and low-intensity regions due to stretched-out signal diluted into surrounding voxels (empty arrows). The use of a B0 field map enables a sound signal reconstruction in stretched areas. However, we observe the same ringing artifacts in previously compressed areas as in Andersson et al. (2003). These artifacts, that originate from the ill-posed problem of recovering true intensity of two voxels that has been pilled-up into a single one, can only be solved by the acquisition of b0 volumes with RPE scheme (FSb0RPE and FSfullRPE pipelines).

**FIGURE 3:**
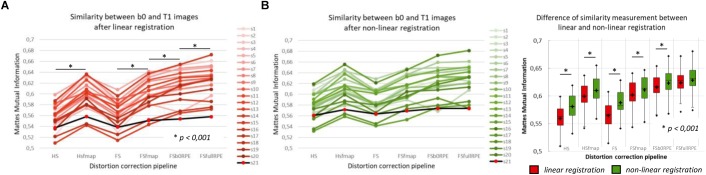
Quantitative assessment of distortion correction methods using the similarity between diffusion and structural images. The Mattes Mutual Information was computed as a similarity measure between the T1w image and the average b0 image registered into structural space, using affine transformation **(A)** or non-linear transformation **(B left)**. The black line with red dots corresponds to the clinical dataset. Student’s paired *t*-tests were performed to compare the registration accuracy between linear and non-linear transformation for each pipeline separately **(B right)**. The significant differences attest to the residual distortions corrected with the non-linear registration. Significance threshold was set to 0.001 to account for multiple comparisons.

The MMI (Figure 3A) quantitatively reflects these observations with a significant effect of preprocessing strategy on the similarity between b0 images and T1w images [*F*(5,19) = 244.7, $\eta_{p}^{2}$ = 0.93, *p* < 0.0001]. In particular, we found that the information obtained from either a field map or RPE images significantly improves the similarity score indicating that such corrected images get closer to the subject’s true anatomy. *Post hoc* tests revealed that the use of FSb0RPE yielded better results than the use of a field map (*t*_HSfmap__<__FSb0RPE_ = 7.9 and *t*_FSfmap__<__FSb0RPE_ = 6.6, *p* < 0.0001). In general, the best similarity score was obtained using the FSfullRPE pipeline (*t*_FSb0RPE__<__FSfullRPE_ = 5.5, *p* < 0.0001), where susceptibility and eddy-current distortions are estimated and corrected simultaneously with a single deformation field. We notice that the correction of movements and eddy-currents using FS did not improve the registration compared to the ECCAR method with HS pipeline. This is expected since the non-weighted diffusion volumes, used in the similarity measurement, are not impacted by eddy-currents distortions. Yet, while both methods seem to equally perform in motion correction, we observed a high decrease in the similarity measurement when substantive subject motion is not corrected (see Supplementary Material S3).

In a second analysis, we computed the similarity metric between images non-linearly registered to evaluate the performance of the non-linear transformation in handling residual geometric distortions. The non-linear registration has been used in several studies to correct for susceptibility-induced distortions (Kybic et al., 2000; Merhof et al., 2007; Tao et al., 2009; Bhushan et al., 2016). However, the generalization of registration parameters setting across subjects is challenging and is highly sensitive to the type of anatomical sequence used or the presence of lesion (Albi et al., 2018). Here, we only evaluated its interest as a complement to the initial pipeline to improve alignment between anatomical and diffusion spaces. After the initial linear registration, the high SNR b0 image was non-linearly registered into the T1w image referential using *‘reg f3d’* as described in Section “Structural to Diffusion Space Registration.” A first visual assessment in Figure 2B shows that the alignment of the b0 images with the gray-white interface boundary is improved for HS and FS pipelines (green arrows). Using Student’s paired *t*-test, we quantified the improvement of this method with respect to the linear registration (see Figure 3B). We show that the non-linear transformation significantly improves the similarity score between the b0 and the T1w images except for the FSfullRPE pipeline, where results were not different. This effect is particularly visible for pipelines which correct only for eddy-currents distortions (HS and FS: *t*_HS_ = -12.77 and *t*_FS_ = -15.13 respectively, *p* < 0.0001). These results corroborate the fact that the non-linear transformation, based on local deformations of voxels, can partly corrects for residual geometric distortions. This is in line with the observation of Calhoun et al. (2017) who used the T1 MNI template as reference image rather than the individual T1 image. Interestingly, the difference is not significant after Bonferroni correction when using the FSfullRPE pipeline (*t*_FSfullRPE_ = -2.638, *p* = 0.016), suggesting that this method yielded optimal correction with least residual distortions left.

Supplemental analyses were performed (results not presented in this article) to ensure that the effect of non-linear registration was not driven by potential residual deformations between diffusion and T1 images caused by differences of gradient non-linearities due to the change in position between the two sessions.

With the clinical data, we observed similar variations of the MMI between pipelines but with lower amplitude. The non-linear transformation also improved the similarity score. Moreover, we found that the *b*-value had no impact on the similarity metric (see Supplementary Material S5).

### Experiment 2: Impact on Diffusivity Measurements: Global Differences

In this section, we investigated the impact of each of the 6 preprocessing pipelines on the diffusion signal modeling. For this purpose, the tensor model was estimated using the weighted least square method from Dipy (Garyfallidis et al., 2014) as described in Section “Diffusion Model Estimation and Tractography,” on the diffusion data corrected through the six preprocessing pipelines. From the tensor model, we extracted two quantitative (TFE, mean dispersion index) and two qualitative (mean diffusivity, fractional anisotropy) metrics (Kim et al., 2006) to compare the quality of tensor estimation with respect to the distortion correction method.

The tensor-fitting error (TFE) used as a measure of the goodness-of-fit of the model (Papadakis et al., 2003) was defined in each voxel with

$$TFE=\sum_{i=1}^{N}{\left(S_{mi}-S_{fi}\right)}^{2}\;\;\;\;\;\;\;\;\;\;\;\;\;\;\;\;\;\left(2\right)$$

where *S_mi_* is the measured signal, *S_fi_* is the fitted signal and N the number of diffusion-weighted volumes. A low TFE, i.e., more signal information fitted in the tensor calculation, is expected with better pre-processing.

The mean dispersion index (MDI) (Basser and Pajevic, 2000) indicates the directional variations of the principal eigenvector in the neighborhood (S) of each voxel:

$$MDI=\frac{1}{n(S)}\sum_{x\in S}\sqrt{\frac{\lambda_{2}+\lambda_{3}}{2\lambda_{1}}}\;\;\;\;\;\;\;\;\;\;\;\;\;\;\;\;\;\left(3\right)$$

where *λ_i = 1,2,3_* are eigenvalues of the mean dyadic tensor derived from principal eigenvectors of the tensor in every voxels x of the neighborhood. This value was extracted for each voxel in the white-matter by considering a neighborhood of two voxels along each axis. Pre-processing should lower the dispersion of the tensor and thus reduce the MDI.

We also evaluated the impact of preprocessing pipelines on the usual diffusion indices of mean diffusivity (MD):

$$MD=\frac{\lambda_{1}+\lambda_{2}+\lambda_{3}}{3}\;\;\;\;\;\;\;\;\;\;\;\;\;\;\;\;\;\left(4\right)$$

and fractional anisotropy (FA):

$$FA=\sqrt{\frac{1}{2}}\frac{\sqrt{{\left(\lambda_{1}-\lambda_{2}\right)}^{2}+{\left(\lambda_{2}-\lambda_{3}\right)}^{2}+{\left(\lambda_{3}-\lambda_{1}\right)}^{2}}}{\sqrt{\lambda_{1}^{2}+\lambda_{2}^{2}+\lambda_{3}^{2}}}\;\;\;\;\;\;\;\;\;\;\;\;\;\;\;\;\;\left(5\right)$$

Values were averaged across all white-matter voxels. The effect of preprocessing on these four indices was assessed using the same statistical analysis as for the MMI. The significance threshold was set to 0.0008 [0.05/(15pairs × 4indices)].

**FIGURE 4:**
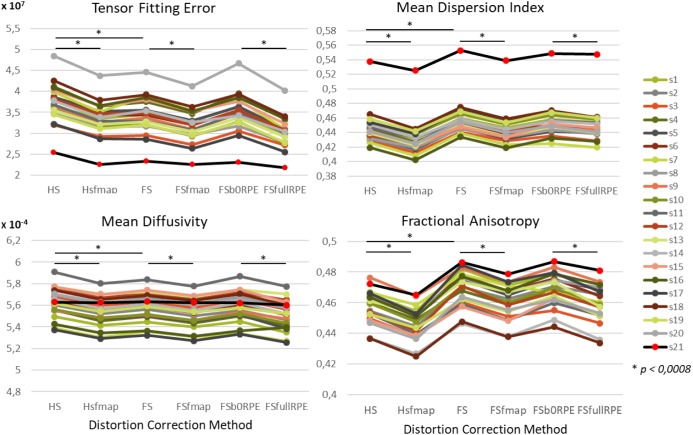
Effect of preprocessing pipelines on the values of four tensor-derived indices TFE, MDI, MD and FA in the white matter. The values of each index were averaged across all white-matter voxels. For every subject represented with different colors for the HCP dataset and in black (red dots) for the clinical dataset, the mean values are plotted as a function of the preprocessing pipeline used to correct distortions.

We found an important reduction of the inter-individual variability in all the tensor-derived indices between uncorrected and corrected data (Supplementary Figure S3). Yet, as illustrated in Figure 4, we observed that the choice of preprocessing pipeline result in significant variations in the values of all tensor-derived indices [*F*_TFE_(5,19) = 390.62, $\eta_{p}^{2}$ = 0.95, *p* < 0.0001; *F*_MDI_(5,19) = 348.88, $\eta_{p}^{2}$ = 0.95, *p* < 0.0001; *F*_FA_(5,19) = 200.3, $\eta_{p}^{2}$ = 0.91, *p* < 0.0001; *F*_MD_(5,19) = 178.7, $\eta_{p}^{2}$ = 0.90, *p* < 0.0001], with a particularly high consistency across individuals. *Post hoc* analyses (see statistics in Table 2) revealed significant differences between eddy-current correction methods, showing decreased TFE and MD and increased MDI and FA for data corrected by *eddy* (FS, FSfmap, FSb0RPE, and FSfullRPE) compared to ECCAR (HS and HSfmap). Second, we found that, for all subjects, the four metrics were jointly decreased by the additional correction of susceptibility-induced distortions using a field map image. On the contrary, the additional correction of susceptibility-induced distortions using FSb0RPE (compared to FS pipeline) did not yield significant differences in any indices. Thus, the effect of field map-based correction could be attributed to a smoothing effect induced by the second resampling involved in this method, rather than an actual distortion correction. Finally, all the tensor-derived indices were significantly reduced when using FSfullRPE compared to FSb0RPE. These results suggest that susceptibility-induced distortion correction has no impact on the global tensor metrics, but only the method used to correct for motion and eddy-currents do.

**Table 2:** Statistical results of the *post hoc* analyses to compare the impact of distortion correction pipelines on tensor-derived indices, using Student’s paired samples *t*-tests.

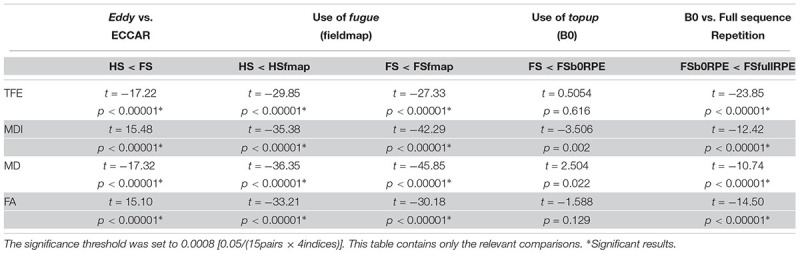

The clinical data presented similar variations for all tensor-derived indices, but with a lower TFE and higher MDI. In addition, we found that the *b*-value had an impact on each index: TFE and MDI increased with *b*-values, and MD and FA were largely decreased with higher b-values (see Supplementary Material S6).

### Experiment 3: Impact on Diffusivity Measurements: Local Differences

**FIGURE 5:**
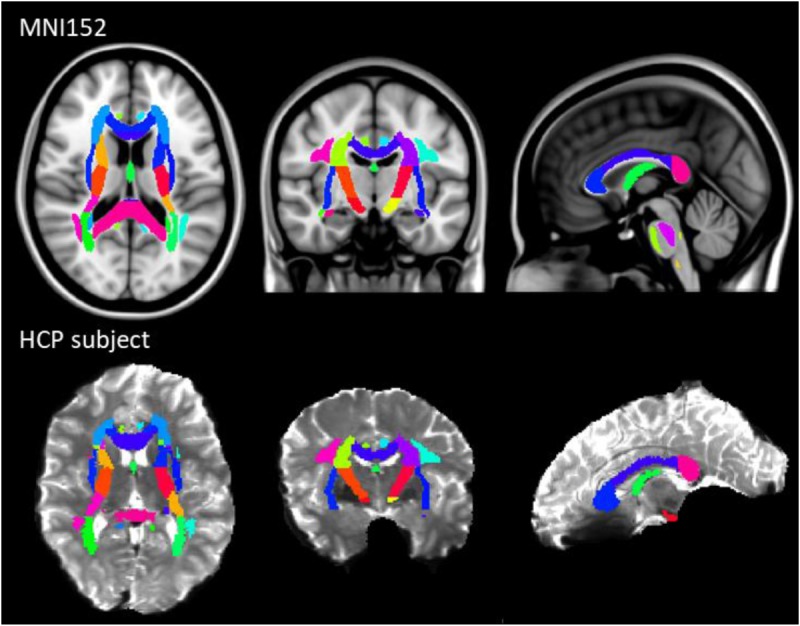
JHU-ICBM-DTI-48 white-matter atlas displayed in the MNI standard coordinate space **(top)** and registered into the diffusion space of an individual after *ECCAR* correction (HS pipeline) **(bottom)**. 38 out of the 48 ROIs were included in the image. Note that even in the case of the simplest correction pipeline, the central brain regions are properly aligned with the subject’s anatomy.

**FIGURE 6:**
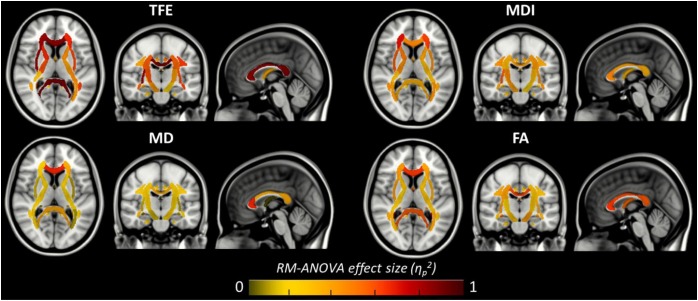
Amplitude of the local main effect of preprocessing pipelines on the four tensor-derived indices TFE, MDI, MD, and FA. For each index, the mean values were computed across the 38 regions of the JHU-ICBM-DTI-48 atlas and compared between the six preprocessing pipelines using a repeated measures ANOVA. Effect sizes (partial eta-squared) are overlaid on the MNI-152 standard brain. A significant effect of preprocessing pipelines was found in regions with partial eta-squared above 0.19 (*p* < 0.001 to account for multiple comparisons across the 38 ROIs).

**FIGURE 7:**
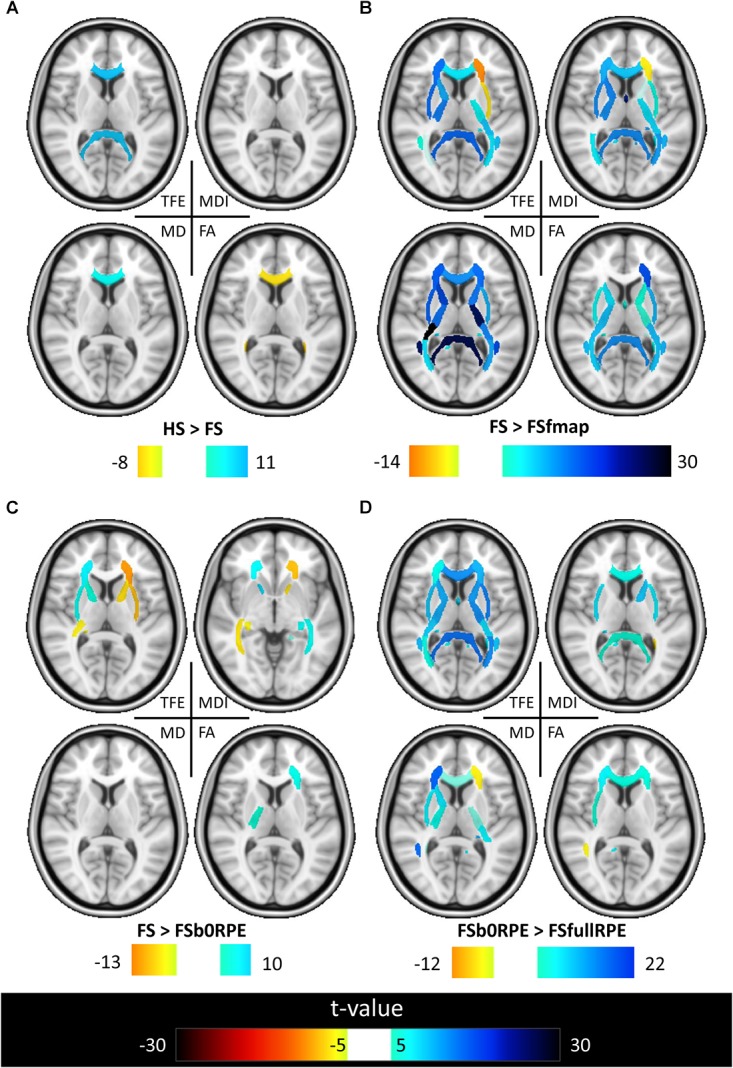
Results of the *post hoc* analyses on the local effect of preprocessing methods on tensor-derived indices. *Post hoc* paired *t*-tests were conducted in all ROIs. Results are shown for the comparison between **(A)** HS and FS pipelines to assess the influence of eddy-current correction method, **(B)** FSfmap and FS pipelines to assess the influence of geometric distortion correction using a B0 field map, **(C)** FSb0RPE and FS pipelines to assess the influence of geometric distortion correction using a b0 with RPE, **(D)** FSfullRPE and FSb0RPE pipelines to compare the influence of using b0 versus the full sequence with RPE. Only regions showing significant differences are shown (|*t*| > 5, *p* < 8.10^-5^ corrected for multiple comparisons).

The results of previous section could be difficult to interpret for several reasons. First, the comparison between pairs of pipelines can be hampered by a number of confounding factors inherent to the correction methods. Indeed, apart from the distortion correction performances, the methods differ in the number of resampling steps applied to the data (two for HSfmap and FSfmap pipelines, one for the others), in the use of intensity correction, and in the level of SNR in corrected images. Second, the tensor-derived indices should constitute reliable metrics in regions where the tensor is an appropriate model of the diffusion signal, which excludes regions with crossing fibers and superficial white-matter. Thus, in this section we investigated spatial heterogeneity in the differences observed on the tensor-derived indices in deep white-matter regions with single fiber direction. Indeed, differences caused by interpolation and resampling should have spatially homogeneous effects in the brain whereas differences caused by the performance of the distortion correction should affect preferentially regions closer to susceptibility gradients or adjacent to areas with distinct tissue architecture. For this purpose, we non-linearly aligned the Johns Hopkins University DTI-based white-matter atlas (JHU-ICBM-DTI-48) (Mori et al., 2005) first into the structural space of each individual using *‘fnirt’* (which provides preconfigured parameters for MNI standard to T1 image registration), and then into the diffusion space using the non-linear registration of Niftyreg, as described in Section “Structural to Diffusion Space Registration.” After registration, all ROIs were binarized using a threshold at 0.5 to prevent overlapping while keeping large enough ROIs to capture tracts (see next section). From the 48 original labels, 10 (mostly included in the brain stem) fell out of the field of view and were excluded from the analysis. Results for one subject are illustrated on Figure 5. In the remaining 38 regions, we computed the average TFE, MDI, FA, and MD and compared the distortion pipelines in the same way as in the previous section (see RM-ANOVA results in Figure 6). Results of the comparison between four pairs of pipelines are illustrated in Figure 7, where only regions showing a statistically significant difference are shown (*p* < 8.10^-5^ corrected for multiple comparisons).

Figure 6 shows that, although far from the air/bones interfaces, most of these central regions are significantly impacted by the choice of distortion correction pipelines. The effect size of RM-ANOVA is particularly high for the local TFE index (above 0.5 in 50% of the regions). *Post hoc* paired *t*-tests revealed that the eddy-currents correction methods (HS versus FS pipelines) has a significant influence on the tensor fitting quality in the corpus callosum (genu, body, and splenium), the best fit obtained using FS, with a significant impact on FA and MD indices (see Figure 7A). Second, we found that the influence of field map-based correction was highly homogeneous for all indices with significant reduction between FS and FSfmap pipelines in respectively 71, 73, 89, and 87% of ROIs for TFE, MDI, MD and FA (see Figure 7B). This result supports the hypothesis of a smoothing effect due to the double resampling of the image. Conversely, we found that the additional correction of susceptibility-induced distortions using FSb0RPE (compared to FS pipeline) yielded spatially heterogeneous differences on local TFE, MDI, and FA, with 47, 29, 8% of ROIs respectively affected (see Figure 7C). Interestingly, we can observe a reverse symmetry in the effect size, which reminds the symmetrical signal compression and dilution in both hemispheres due to susceptibility artifacts. Lastly, the use of FSfullRPE compared to FSb0RPE resulted in a homogeneous increase of tensor fitting quality (92% of ROIs for TFE) but a spatially heterogeneous effect for all other tensor-derived metrics with significant differences in 37, 50, and 34% of ROIs in MDI, MD and FA respectively (see Figure 7D). The latter suggests that this is not an effect of resampling as with the fieldmap method. Instead, the significant decrease of Mean Dispersion Index supports a local improvement of the tensor fitting quality. Then, the homogeneous decrease of TFE could be attributed to the higher SNR in images corrected with FSfullRPE.

### Experiment 4: Impact of Preprocessing Methods on Tract Reconstruction

In this section, we evaluated the influence of preprocessing pipelines on the trajectory of six well-known fascicles of different sizes. Tracts reconstruction was performed using the global Gibbs tracking algorithm (Reisert et al., 2011), as described in Section “Diffusion Model Estimation and Tractography.” The interest of this method in our experiment is many-fold. First, Global tractography principle is based on optimization processes that reconstruct all fibers at the same time, avoiding the need of seeding strategies as opposed to step-by-step approaches which has been found to modulate the shape and density of fibers within fascicles (Girard et al., 2014). In our case, the use of a seeding strategy would prevent any comparison of fiber bundle trajectories between differently pre-processed – thus non-aligned – brains. Second, Global tractography has been found more robust to local errors in the fiber orientation estimation (Reisert et al., 2011; Mangin et al., 2013). In particular, the global Gibbs tractography (Reisert et al., 2011) was found to outperform deterministic and probabilistic methods in various connectivity metrics (Fillard et al., 2011; Neher et al., 2015), in particular showing higher ability to detect valid bundles, higher bundle coverage, and less prematurely ending fibers (Christiaens et al., 2015). Finally, this method was chosen for the valuable compromise between computational time, tractogram quality, and file sizes for a whole-brain tractography (20 subjects with 6 preprocessing pipelines led to 120 tractograms). Global tractography was performed using the default parameters for a dense reconstruction (3.108 iterations, 50 steps, starting/stopping T° = 0.1/0.001, σ = 1 mm, l = 3 mm, *w* = 0.07). The whole-brain tractogram was computed using the white-matter mask as constraint, after being registered into the diffusion space. From each individual whole-brain tractogram we extracted the following fascicles: the cortico-spinal tract, the corpus callosum, the superior longitudinal fascicle, the cingulum, the uncinate and the fornix fascicles. These tracts were chosen because they pass through the most distorted areas, cover the three spatial directions, and can be identified for every subject. They were extracted using ROIs of the JHU-ICBM-DTI-48 atlas either as way-points or as exclusion-points following the recommendations described in (Catani and Thiebaut de Schotten, 2008). The labels used are detailed in Table 3. To compare the impact of the different preprocessing pipelines on tractography we analyzed the spatial variance of each fascicle as in (Irfanoglu et al., 2012). This measurement first described in Lazar and Alexander (2005) quantifies the spatial dispersion of the fibers trajectory with the distance from the seed. It is obtained by considering all voxels in the fascicle that are at a certain distance (in voxels) from the seed (here the way-point mask), and computing the covariance matrix of these voxels’ coordinates, weighted by the density of fibers crossing them. For a full description of the spatial signature of the tracts we extracted the spatial variance along the X, Y, Z axes, given by the diagonal elements of the covariance matrix, as well as the spatial variance along the principal mode, given by the primary eigenvalue. The former corresponds to a description of the 3D shape of the fascicles and can be used to measure their similarity across subjects or across preprocessing pipelines. The latter can be interpreted as a measure of the spatial dispersion of the tract to assess the impact of distortion correction methods. We plotted the spatial variances as functions of the absolute distance to the seed, for each subject. The curves were smoothed by convolution, over a sliding window of size 5, and we computed the average curve across subjects. These tract signatures were compared between preprocessing pipelines by performing a RM-ANOVA on the area under the curve (AUC).

**Table 3:** Labels of the JHU-ICBM-DTI-48 atlas used either as way-points or exclusion-points to extract the fascicles from the “whole brain” tractograms.

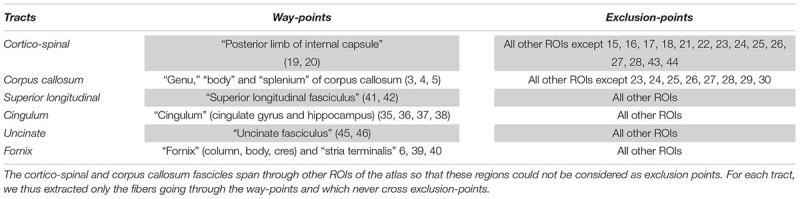

**FIGURE 8:**
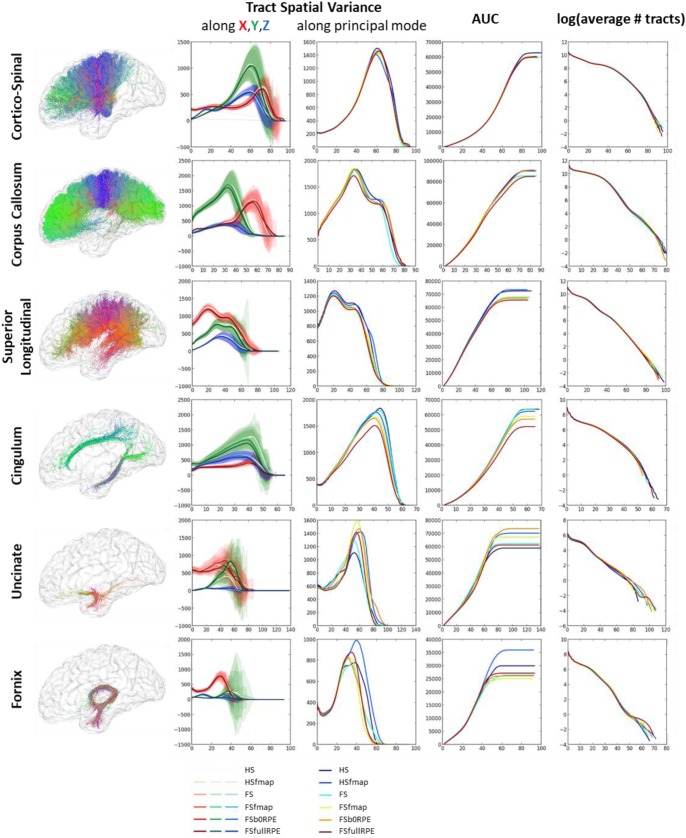
Results of the global tractography reconstruction and spatial dispersion analysis. Six fascicles were extracted from the whole brain tractograms using ROIs of the JHU white-matter atlas as way-points and exclusion-points. **(First column)** On the left are illustrated these fascicles for one subject after data preprocessing using the FSfullRPE pipeline. **(Second column)** On the graphs are plotted the tract signatures for the six pipelines, that is the mean spatial variance of tracts (and standard deviation across subjects) along the *X, Y*, and *Z* axes as a function of the absolute distance to the seed. **(Third column)** The graphs show the spatial dispersion of the tracts, that is the mean spatial variance of tracts along the principal mode. **(Fourth column)** The graphs show the cumulative area under the curve of the spatial variance along the principal mode. It represents the amount of spatial dispersion from the seed. A RM-ANOVA was conducted on the total AUC values to compare the spatial variance between pipelines. **(Fifth column)** The log of the number of fibers is plotted at each distance to the seed.

Figure 8 shows that each tract has a specific spatial signature along the *X, Y, Z* axes. For instance, the cortico-spinal tract showed a distal higher variance along the *Y* axis while the superior longitudinal tract showed a proximal higher variance along the *X* axis. These signatures looked highly similar across subjects and across preprocessing pipelines (as seen on the variance plot of the second column of Figure 8) although we observed more variability for smaller fascicles such as the cingulum, the uncinate and the fornix. We found a significant influence of the preprocessing strategy on the tract spatial variance along the principal mode (third column) for the corpus callosum, the superior longitudinal and the cingulum fascicles [respectively *F*(5,19) = 3.59, $\eta_{p}^{2}$ = 0.16, *p* = 0.005; *F*(5,19) = 7.49, $\eta_{p}^{2}$ = 0.28, *p* < 0.00001; and *F*(5,19) = 8.69, $\eta_{p}^{2}$ = 0.31, *p* < 0.00001] with better scores obtained for the FSfullRPE pipeline, and a tendency for the cortico-spinal fascicle [*F*(5,19) = 2.62, $\eta_{p}^{2}$ = 0.12, *p* = 0.029] with higher spatial variance observed for the HS pipeline compared to others. The AUC curves (fourth column) indicate the distance from the seed at which the signatures start to differ. The last curves (fifth column) show that the number of fibers does not differ between pipelines, indicating that reductions of variance are not due to a loss of fibers.

## Discussion

In this article, we studied the influence of preprocessing distortion correction pipelines on diffusivity metrics and tractography measurements. For this purpose, we developed the *Diffuse* toolbox for DWI data processing which provides, in a guided user interface, the adapted preprocessing pipeline according to the data acquisition settings. Six different distortion correction pipelines are available, compatible with most acquisition type from clinical to research context. Two diffusion models and three tractography algorithms constitute the post-processing steps. Embedded in the BrainVISA open-source platform, the toolbox comes with an automatic indexation of data into a database organization as well as a visualization tool, and an access to processed anatomical data. This software configuration was well suited to investigate the impact of preprocessing methods on diffusivity measurements and tractography.

To our knowledge, the previous work that is most similar to our study is Yamada et al. (2014) where authors compared the following 4 pipelines: ‘eddy_correct’ using trilinear interpolation; ‘eddy_correct’ using spline interpolation; ‘eddy’ combined with ‘topup’ on 60 diffusion gradients and 2 non-diffusion volumes with RPE, equivalent to our FSb0RPE pipeline; and ‘eddy’ combined with ‘topup’ on 30 diffusion gradients repeated with RPE, equivalent to our FSfullRPE pipeline. To assess the differences between these 4 pipelines, authors compared the FA values within the white-matter skeleton, assuming that higher FA should be associated to better distortion correction. Indeed, increased FA could result from restricted perpendicular diffusivity, facilitated parallel diffusivity, or some combination of the two, reflecting a reorganization in tissue structure. However, in our work, we also observed that diffusivity metrics can be affected by other cofounding factors such as interpolation and smoothing effects. The major contributions of our study are:

- We compared quantitatively the impact of the distortion correction using a field map in place of ‘topup’.
- We quantified the correction quality with a similarity metric between DWI and T1
- We quantified the quality of tensor fitting with TFE and MDI indices.
- We quantified the impact on tract spatial dispersion.

### The Most Performant Acquisition/Preprocessing Choice

From the quantitative analyses of this study, we were able to sort the six pre-processing pipelines regarding the following performance criteria: ability to recover brain’s true geometry (through the MMI index); tensor fitting quality (through the TFE index) and tract spatial variance. As expected, for all these quantitative indices, the best score was obtained with the FSfullRPE pipeline, that is when all diffusion gradients are repeated twice with RPE. Importantly, we showed that the FSfullRPE pipeline yielded the best similarity results, i.e., the geometry of the brain was quasi completely recovered, as shown by the equal performance of linear registration compared to non-linear registration. In previous studies, this pipeline has also been shown to outperform the ‘*eddy_correct*’ tool, in terms of eddy-current distortion correction, for *b*-values between 1500 to 7000 s/mm^2^ (Andersson and Sotiropoulos, 2016). In terms of susceptibility-induced distortion correction, the ‘topup’ tool has been shown to outperform the use of a field map acquisition (Andersson et al., 2003). Compared to uncorrected data, this distortion correction pipeline yielded higher FA values in the white matter as found in Yamada et al. (2014) and lower MD values.

In the following, we will discuss the valuable interest of other acquisition/pipeline choices, from the minimum requirements (smaller set of acquired images and HS pipeline) to this optimal preprocessing pipeline that requires a large number of acquisitions, though at the cost of twice longer scan time.

### Motion Correction Reduces Inter-Individual Variability in Tensor Metrics

Our results on uncorrected data showed that the similarity between b0 and T1 images (Supplementary Material S3) as well as the tensor-derived metrics (Supplementary Material S4) were highly impacted by the subject movements. Interestingly, we found that every preprocessing pipeline was able to reduce the inter-individual variability due to a difference in head movements during the scan. This finding emphasizes the importance of motion correction to improve the tensor model estimation.

This should be particularly relevant when comparing healthy subjects and patients who are more likely to move in the scanner (Yendiki et al., 2014; Taylor et al., 2016). Note, however, that we did not address the issue of signal dropout due to fast “bulk” motion of the subject during the acquisition of a volume. This artifact is likely to occur in a clinical context where patients and children are usually less compliant and more subject to discomfort in the scanner. It can induce important signal loss in several slices that can have dramatic consequences on post-processing and diffusivity measurements (Roalf et al., 2016; Baum et al., 2018). Several methods have been developed to detect and remove (Oguz et al., 2014) or correct (Chang et al., 2005; Farzinfar et al., 2013; Andersson et al., 2016, 2017) this erroneous slices. Once motion correction is performed, we observe a high inter-subject consistency in the variation of MMI as well as tensor derived metrics between the six preprocessing pipelines. This observation reinforces the strength of variations between pre-processing pipelines that we will discuss hereafter.

### On the Interest of Eddy-Current Distortion Correction

When considering all the subjects, with and without important head movements, we observed a general (not only for subjects who presented substantial movement) and substantial reduction of TFE and MD, and an increase of MDI and FA in the white matter (see Supplementary Material S4). A similar increase of FA was observed in Yamada et al. (2014) with the use of *‘eddy_correct’* with spline interpolation. This result, found for both ECCAR (HS) and ‘*eddy’* (FS) methods in our study highlights the importance of this step in the preprocessing pipeline. Yet, we found significant differences depending on the method used.

#### On the Benefit of Using a Full-Sphere Sampling Scheme

Our results showed an even better tensor fitting quality when using *‘eddy’* (FS, FSfmap, FSb0RPE, FSfullRPE pipelines; full sphere sampling scheme) compared to the ECCAR method (HS and HSfmap pipelines; HS sampling scheme). Note that the HCP data were acquired with strong gradients (up to 100 mT/m), high *b*-values (up to 3000 s/mm^2^) and high spatial resolution. In this “research-type” context, images were strongly affected by susceptibility and eddy currents deformations and it is not surprising that the use of a first order affine transform (ECCAR) rather than a high order model (*eddy*) results in a poor alignment between successive volumes, which in turn can affect the quality of the diffusion tensor estimation. Similar conclusions were drawn from the study of Jezzard et al. (1998), where authors performed an in-depth comparison between *‘eddy’* and *‘eddy_correct’* tools, from which is derived the ECCAR method. In Graham et al. (2016), authors confirmed the higher performance of ‘*eddy*’ over *‘eddy_correct’* on realistic numerical simulations of DWI with distortions. Indeed, the performance of eddy-current correction using the *‘eddy_correct’* method was found to depend on the *b*-value and/or SNR of DWI data (Nilsson et al., 2015; Graham et al., 2016), with lower registration quality for higher *b*-values. In our case, ECCAR uses a two-step approach to register the DWI volumes, first to the closest b0 volume and second to the first acquired one, in combination to the use of mutual information as cost function. Although this might greatly improve the registration quality compared to *‘eddy_correct’*, this is not sufficient to properly correct for eddy-current and motion in high *b*-value data. It would be interesting to further investigate our metrics on data with lower *b*-values and fewer gradient directions, in addressed in Graham et al. (2016) where authors evaluated the robustness of ‘*eddy*’ with *in silico* simulations.

Note that we purposely chose to use the same resampling scheme with spline interpolation in both pipelines to avoid confounding effects. Indeed, interpolation techniques used to resample data are known to play a critical role in the final quality of the image and particularly in the robustness of the registration algorithm (Mahmoudzadeh and Kashou, 2013). Notably, the trilinear interpolation, often used as default parameter, usually results in less intensity errors but more blurring in the image than other methods. Here, we cautiously employed the same interpolation method (spline) in all pipelines. However, further investigations showed that the use of trilinear interpolation for ECCAR had the effect to increase TFE and reduce MDI, FA, and MD. This results corroborates the alternative decreases or increases of FA observed in Yamada et al. (2014) when using respectively trilinear or spline interpolation in *‘eddy_correct.’*

#### Eddy-Current Distortions Also Affect Central White-Matter Regions

ROI-based analysis revealed that the improvement of the tensor fitting is localized in the corpus callosum and is accompanied by a decrease of MD and an increase of FA mostly in the genu of the corpus callosum. Two reasons could explain this finding. First, the corpus callosum is defined by a high anisotropy and a high directionality of the diffusivity. Thus, this area is likely to be sensitive to a small difference in the tensor estimation. Second, a poor alignment of successive volumes could impact differently the tensor model in regions surrounded by different white-matter architectures. Indeed, a **residual shift in voxels position** often leads to a characteristic rim of high anisotropic voxels at the edge of the brain (Alexander et al., 1997; Jones and Cercignani, 2010). However, while this outside effect is visually easy to detect, a similar effect can happen at the intersection between distinct tissue types or micro-structural architectures such as white-matter and CSF (Jezzard et al., 1998). For instance, as observed in local analyses for FA and MD, the genu of the corpus callosum is in a brain region that is highly prone to geometric distortions and is adjacent to the lateral ventricles. Likewise, in the literature, the influence of eddy-current distortion correction on the diffusivity indices has often been reported differently depending on the regions studied. For instance, previous visual observations of fractional anisotropy maps showed sharper contours and reduced blurring after eddy-current corrections using gradients with reversed polarity (Alexander et al., 1997; Bodammer et al., 2004), compared to no correction. Other quantitative studies found increased FA in corrected data using affine registration (Rohde et al., 2004), as well as using FS-equivalent method (Shen et al., 2004), in several regions which were not visible in the anisotropy maps of uncorrected data. However, in Kim et al. (2006), authors found a decrease of FA for several correction methods in the uncinate and corpus callosum tracts. Finally, in Rohde et al. (2004), an artificial increase of anisotropy in the left-right orientation, in isotropic regions such as gray-matter, was reduced after correction, while MD was not affected.

### On the Interest of Susceptibility-Induced Distortion Correction

The first experiment clearly demonstrates the ability of susceptibility-induced distortion corrections to recover the brain’s true geometry. In line with Cusack et al. (2003) and Tao et al. (2009) we found that the use of a field map brings significant improvement in the registration accuracy between DWI and T1w data. In Cusack et al. (2003), authors ascertained that this difference did not originate from the slight smoothing produced by the resampling procedure. Here, the significant improvement also measured with the FSb0RPE compared to the FS pipeline, which both use the same resampling procedure, further supports the benefit of susceptibility-induced distortion correction. However, we observed major differences between the use of a field-map and the use of a b0 volume to correct for these distortions.

#### Reversed b0 Volume Outperforms Field-Map

First it should be noted that the field map images were not acquired during the same session as the diffusion images. Thus, a change in head position in the scanner probably led to slight variations in the field map induced by the interaction between shimming and gradient non-linearities. As a consequence, our field map-based correction shows probably lower performance than it should if the field map was acquired during the dMRI session. Second, compared to the use of a field map, the advantage of FSb0RPE is two-fold. In addition to the improved registration accuracy (results Experiment 1), with a more realistic signal reconstruction in stretched and compressed areas as seen in Figure 2A, the FSb0RPE pipeline (as well as the FSfullRPE) combines both motion, eddy-current and susceptibility distortions in a single distortion field to correct simultaneously for all these artifacts (Andersson and Sotiropoulos, 2016). Conversely, the field map-based correction is performed as a second step, involving a second resampling and interpolation of signal intensity which is likely to induce smoothing in the corrected images (Wang et al., 2017).

Indeed, ROI-based analyses showed that, compared to HS and FS pipeline, the additional use of a field map resulted in a highly homogeneous reduction of all tensor-derived metrics, while we expected the effect to be higher in regions prone to severe geometric artifacts, as reported in Wu et al. (2008). To understand this artificial decrease of tensor-derived metrics, one has to understand the effects that a 3D smoothing has on the 4th dimension of DWI data (i.e., across gradient directions). In fact, we can imagine that the smoothing would flatten the ellipsoid of the tensor model by removing high frequency fluctuations in the signal. As a consequence, one can expect that the tensor model would give better fitting performance and the TFE as defined by the equation (2) should be reduced. Besides, when considering only white-matter voxels where the signal is highly anisotropic, i.e., low intensity signal in a given gradient direction and high intensity in the others, the smoothing should flatten the signal of the voxel across volumes which explains the reduced FA and MD. Finally, the differences between neighboring voxels which diffuse in different directions are also flattened, thus decreasing the local variation of the tensor orientation represented by the MDI.

Conversely, ROI-based analyses revealed a local influence of the distortion correction using b0 volumes with RPE compared to the FS pipeline. Interestingly these results concern regions closest to the frontal and temporal lobes, with symmetric effects for TFE and MDI, which echoes the left-right orientation of geometric distortions. Note that this symmetry induces a compensation which might account for the null global effect. Importantly, the methodological difference between FS and FSb0RPE pipelines is that, in the latter, the distortion field used in *eddy* to correct data also includes the susceptibility-induced distortions estimated with *topup*. Besides that, both methods use the same interpolation procedure and the same intensity reconstruction (by Jacobian modulation).

In Cusack et al. (2003), authors highlight another disadvantage of B0 field map acquisition which does not capture the interaction between distortion field and subject movements during the scan which could introduce additional variations across subjects. In particular, this should be kept in mind when comparing different types of population such as healthy volunteers and patients or children who are more prone to motion. These findings emphasizes the benefits of acquiring a single b0 volume with RPE instead of a double-echo field map sequence, for an acquisition time of respectively a few seconds and around 2 min, a substantial difference in the context of clinical acquisitions (Treiber et al., 2016). Note also that equivalent co-registration quality with T1 was found for FSb0RPE and FSfullRPE pipelines after non-linear registration.

#### Differences Between FSb0RPE and FSfullRPE

The methodological difference between FSb0RPE and FSfullRPE lies in the repetition of all diffusion weighted volumes twice in the latter pipeline. The interest is twofold. First, every pair of volumes with opposed phase-encoding directions are averaged, yielding the same final number of volumes as for the other pipelines. This doubles the amount of information in each voxel, increasing the SNR, which explains the homogeneous decrease of TFE in the white-matter. Indeed, the more information contained in the signal, the easier the tensor model could fit the data. Second, compared to the Jacobian modulation which only account for signal dilution, the least-square restoration provides a better signal reconstruction in compressed areas (Andersson et al., 2003) which could be at the origin of the heterogeneous decrease of MDI, FA and MD. This concords with Yamada et al. (2014), where authors also observed an heterogeneous decrease of FA lateralized in the left hemisphere.

Overall, our results suggest that the correction of susceptibility-induced distortions using RPE scheme provides better tensor fitting performance, in particular with a local influence on tensor-derived metrics. To confirm our hypotheses, it would be interesting to conduct voxel-wise analyses, to test, for instance, the spatial correlation of the four diffusion indices with multiple variables such as signal intensity or local deformation needed to correct for geometric distortions.

### Handling Residual Geometric Distortions

A major outcome of this work is that the quality of the registration between diffusion and structural space mostly depend on the susceptibility-induced distortion correction method. As shown in our results, the remaining geometric distortions are hardly handled by an affine transformation. As a consequence, a misalignment between structural and diffusion space may be present when registering an atlas, a template or a group of subjects together. In order to take into account residual distortions, we highly recommend using a non-linear transformation for inter-subject as well as for intra-subject registration of mask, ROIs or atlases for connectivity purpose (Cusack et al., 2003).

### Impact on Bundle Trajectories

Our results on tract spatial variance revealed very different signatures for each fascicle, supporting the suitability of this metric to quantify the variability in the shape of fiber bundles. However, the tract signatures obtained in this article look highly different from those obtained in Irfanoglu et al. (2012). Several factors could participate in these different results. First, authors in Irfanoglu et al. (2012) used deterministic and probabilistic tractography algorithm, both requiring potentially different parameters such as the number of iterations, stopping criteria and curvature threshold. They also filtered out isolated fibers while we did not, which can explain the higher distal dispersion of tracts in our results. For these reasons, the tractograms of the two articles are hardly comparable. In our study, the consistency of tract signatures across subjects and preprocessing pipelines (Figure 8) illustrates the robustness of the global tractography to reconstruct fascicles regarding inter-individual variability and residual distortions in the image. This observation is particularly true for the largest fascicles of the cortico-spinal, corpus callosum, superior longitudinal, and cingulum pathways.

Despite this robustness, the trajectory of some fascicles is sensitive to the distortion correction strategy. It is the case for the commissural pathway of the corpus callosum and two association pathways, namely the superior longitudinal tract and the cingulum tract which project from the frontal lobe to the temporal lobe. Although this quantitative analysis could not allow a clear distinction between an effect of eddy-current distortions or susceptibility-induced distortions, these two main regions are known to be prone to severe geometrical artifacts due to their proximity to air/bone tissues interfaces. Indeed, as shown in Embleton et al. (2010), the misalignment of voxel-wise fiber orientations in these regions could lead to a premature ending of reconstructed pathways. In the same line, linear and non-linear correction of eddy-current have been found to visually reduce the dispersion of uncinate and corpus callosum tracts, especially in the temporal and frontal parts (Kim et al., 2006). However, the short pathways of uncinate and fornix tracts did not show significant sensitivity to distortion corrections. A possible reason is that the small seeds used to extract these tracts are likely to be subject to higher inter-individual variability and higher registration errors. This could explain why we were not able to distinguish the effect of preprocessing pipelines out of the intrinsic tract variability.

Finally, it should be noted that the global tractography algorithm includes optimization process that takes into account the uncertainty in the DWI data and has been reported to prevent from overfitting (Daducci et al., 2015). Yet, other tractography algorithms such as probabilistic or deterministic tractography might be prone to more important changes due to a higher sensitivity to inter-scan variability.

### Relevance Toward Clinical Data

To relate our observations with the clinical context we reproduced the same experiments on a similar dataset but acquired on a Siemens Prisma 3T MR-system with similar maximum gradient strength and slew rate as for the HCP scanner but using different acquisition settings. In particular, the T1w image had a lower resolution which, as we found, did not alter neither the capacity nor the interest of using non-linear registration to improve the alignment between T1 and diffusion weighted images.

Importantly, the performance differences that we observed between pipelines is still valid for the dataset acquired in a context closer to the clinical environment. Although we found differences between the two datasets, common to all pipelines, as a global shift. For instance, the tensor-fitting quality was highly different from the HCP data which could be attributed to a higher SNR in the images, relative to the lower spatial resolution of DW images and the lower acceleration factor.

With this clinical dataset, we also highlighted the impact of *b*-value on the tensor fitting performance. Indeed, we found a better tensor fitting for lower *b*-values. Also, the FA and MD values measured in the white matter showed an important sensitivity to the *b*-value. This finding is probably related to the amount of SNR as well as the quality of distortion correction. Indeed, lower b-values involve lower gradient amplitudes and thus less eddy-currents. Apart from the amount of distortions, a higher SNR in the images could imply that conventional image registration algorithms perform better (Andersson and Sotiropoulos, 2016). It has been reported in the literature that the amount of noise in the raw image can have an influence on the tensor-derived metrics (Manjón et al., 2013; Hutchinson et al., 2017).

### Limitations and Future Work

One important limitation in our study lies in the choice of regions restrained to the central white-matter for the ROI-based analysis. These brain areas are not the most impacted by geometric distortions. This work should be extended to the rest of the brain, for instance by including regions of interest with superficial white-matter. In particular, it would be interesting to correlate the variation in diffusivity indices to the amplitude of distortions, or the amount of displacement necessary to align each voxel to the structural image. However, this investigation would require using other metrics that are not based on the tensor model, which reliability is limited to regions with single fiber’s direction. More suited models intended to fit the complex white-matter architecture such as NODDI would be more appropriate (Graham et al., 2016) but require specific acquisition settings with multi-shell sampling of gradients, in particular with a “mini-shell” that can model the high-diffusion compartments.

A second limitation is the difficulty to quantify the differences between pipeline’s performance regarding the reconstruction of tracts. Especially, we could not easily reproduce results of previous studies, due to the inability to reproduce the seeds position and the complexity of algorithm parameters settings. One way to overcome these limitations would be to perform similar analyses on numerical phantoms with a known ground truth. Also, further work is necessary to investigate the impact of preprocessing methods on the connectivity measurements between cortical regions. Such analysis would probably be less influenced by outlier fibers that show higher spatial dispersion.

## Conclusion

The aim of this study was to evaluate the impact of different preprocessing pipelines on the quality of corrected data. While most studies try to isolate the cofounding factors coming from acquisition settings, data or processing quality, we instead found interesting to consider the combination of eddy-current and susceptibility-induced distortion corrections into single pipelines dedicated to distinct acquisition contexts. Hence, we could highlight the resulting differences between outcome data and their consecutive diffusivity and tractography measurements. As these pipelines are optimal for different acquisition contexts, our observations will help for both a careful choice of acquisition settings and a precautious interpretation of DWI analysis. In the light of our results, the acquisition of several interspersed b0 volumes plus an additional b0 volume with RPE is highly recommended as default settings, rather than the acquisition of a field-map. Moreover, we highly recommend to use non-linear registration with anatomical images to handle residual distortions. Ideally, acquisition settings should be chosen depending on the study purpose and on the acquisition and processing times that can be afforded depending on the context (e.g., clinical or research). For instance, to compare two different populations, investigators should focus on an efficient motion correction method. However, if effects are expected in regions exposed to magnetic susceptibility differences, such as temporal and frontal lobes, a particular attention should be paid to geometric distortion correction and signal intensity recovery. Besides, investigators should limit the number of resampling steps applied on images to avoid artificial tensor over-fitting. Finally, optimal correction performance can be obtained with FSfullRPE acquisition but at the expense of long acquisition and processing times. A crucial outcome here is that analysis should never be conducted on datasets which underwent distinct preprocessing pipelines. Finally, further investigations should be performed to evaluate the influence of the same pipelines regarding other acquisition settings such as *b*-value, q-space sampling size, and noise reduction, where the correction of eddy-currents should be of major importance.

The Diffuse software toolbox implemented to conduct the present study is available at this link: https://github.com/MecaLab/Brainvisa-Diffuse. It offers an automatic selection of the optimal preprocessing pipeline given the acquired DWI data. It also provides registration with anatomy, local model reconstruction and tractography algorithms. The Diffuse toolbox is embedded in the BrainVISA platform which gives access to volume-based and surface-based anatomical data processing, as well as to an efficient database management.

## Annexe

List of subject IDs as provided by the Human Connectome Project: S1:106319, S2:150625, S3:188751, S4:193441, S5:220721, S6:424939, S7:627852, S8:773257, S9:932554, S10:983773, S11:102513, S12:110613, S13:114621, S14:147030, S15:158843, S16:159946, S17:176441, S18:346137, S19:677766, and S20:942658.

## Ethics Statement

Data were provided by the Human Connectome Project, WU-Minn Consortium (Principal Investigators: David Van Essen and Kamil Ugurbil; 1U54MH091657) funded by the 16 NIH Institutes and Centers that support the NIH Blueprint for Neuroscience Research; and by the McDonnell Center for Systems Neuroscience at Washington University. Clinical data were provided by the MRI Center of the Neuroscience Institute of La Timone, in Marseille. The study received the approval of the Ethics Committee (N_ RCB 2012-A00268-35).

## Author Contributions

LB and AP implemented the data processing toolbox *Diffuse*, processed the HCP dataset through the 6 pipelines and performed the statistical analyses. JS participated to the choice of the optimal database, as well as the tools integrated in the toolbox. JS, CD, and OC brought valuable input to the discussion of the results. LB, AP, and OC released the toolbox publicly on Github. All authors contributed to the preparation and correction of the article, figures, and tables.

## Conflict of Interest Statement

The authors declare that the research was conducted in the absence of any commercial or financial relationships that could be construed as a potential conflict of interest.

## Footnotes

1 https://github.com/MecaLab/Brainvisa-Diffuse

2 http://fsl.fmrib.ox.ac.uk/

3 http://nipy.org/dipy/

4 http://cmictig.cs.ucl.ac.uk/wiki/index.php/NiftyReg

5 http://brainvisa.info

6 RAS+ stands for positive RAS orientation of axes such that X axis goes from left to right, Y axis goes from posterior to anterior and Z axis goes from inferior to superior.

7 http://cmictig.cs.ucl.ac.uk/wiki/index.php/NiftyReg

## Abbreviations

FS: full-sphere
HCP: Human Connectome Project
HS: half-sphere
MMI: Mattes Mutual Information
RPE: Reverse Phase Encoding
TFE: Tensor Fitting Error

## References

[B1] Albi A. Meola A. Zhang F. Kahali P. Rigolo L. Tax C. M. W. (2018). Image registration to compensate for EPI distortion in patients with brain tumors: an evaluation of tract-specific effects. *J. Neuroimaging* 28 173–182. 10.1111/jon.12485 29319208 PMC5844838

[B2] Alexander A. L. Tsuruda J. S. Parker D. L. (1997). Elimination of eddy current artifacts in diffusion-weighted echo-planar images: the use of bipolar gradients. *Magn. Reson. Med.* 38 1016–1021. 10.1002/mrm.1910380623 9402204

[B3] Andersson J. L. R. Graham M. S. Drobnjak I. Zhang H. Filippini N. Bastiani M. (2017). Towards a comprehensive framework for movement and distortion correction of diffusion MR images: within volume movement. *Neuroimage* 152 450–466. 10.1016/j.neuroimage.2017.02.085 28284799 PMC5445723

[B4] Andersson J. L. R. Graham M. S. Zsoldos E. Sotiropoulos S. N. (2016). Incorporating outlier detection and replacement into a non-parametric framework for movement and distortion correction of diffusion MR images. *Neuroimage* 141 556–572. 10.1016/j.neuroimage.2016.06.058 27393418

[B5] Andersson J. L. R. Jenkinson M. Smith S. (2009). Non-linear registration aka Spatial normalisation FMRIB. *Neuroimage* 45 S173–S186. 10.1016/j.neuroimage.2008.10.055 19059349

[B6] Andersson J. L. R. Skare S. Ashburner J. (2003). How to correct susceptibility distortions in spin-echo echo-planar images: application to diffusion tensor imaging. *Neuroimage* 20 870–888. 10.1016/S1053-8119(03)00336-7 14568458

[B7] Andersson J. L. R. Sotiropoulos S. N. (2016). An integrated approach to correction for off-resonance effects and subject movement in diffusion MR imaging. *Neuroimage* 125 1063–1078. 10.1016/j.neuroimage.2015.10.019 26481672 PMC4692656

[B8] Auría A. Daducci A. Thiran J. Wiaux Y. (2015). NeuroImage Structured sparsity for spatially coherent fi bre orientation estimation in diffusion MRI. *Neuroimage* 115 245–255. 10.1016/j.neuroimage.2015.04.049 25944612

[B9] Basser P. J. Pajevic S. (2000). Statistical artifacts in diffusion tensor MRI (DT-MRI) caused by background noise. *Magn. Reson. Med.* 44 41–50. 10.1002/1522-2594 10893520

[B10] Bastiani M. Andersson J. Cordero-Grande L. Murgasova M. Hutter J. Price A. (2019). Automated processing pipeline for neonatal diffusion MRI in the developing Human Connectome Project. *Neuroimage* 185 750–763. 10.1016/j.neuroimage.2018.05.064 29852283 PMC6299258

[B11] Bastiani M. Cottaar M. Dikranian K. Ghosh A. Zhang H. Alexander D. C. (2017). Improved tractography using asymmetric fibre orientation distributions. *Neuroimage* 158 205–218. 10.1016/j.neuroimage.2017.06.050 28669902 PMC6318223

[B12] Baum G. L. Roalf D. R. Cook P. A. Ciric R. Rosen A. F. G. Xia C. (2018). The impact of in-scanner head motion on structural connectivity derived from diffusion MRI. *Neuroimage* 173 275–286. 10.1016/j.neuroimage.2018.02.041 29486323 PMC5911236

[B13] Ben-Amitay S. Jones D. K. Assaf Y. (2012). Motion correction and registration of high b-value diffusion weighted images. *Magn. Reson. Med.* 67 1694–1702. 10.1002/mrm.23186 22183784

[B14] Bhushan C. Haldar J. P. Choi S. Joshia A. Shattuckc D. W. Leahy R. M. (2016). Co-registration and distortion correction of diffusion and anatomical images based on inverse contrast normalization. *Neuroimage* 269–280. 10.1016/j.neuroimage.2015.03.050 25827811 PMC4461504

[B15] Bodammer N. Kaufmann J. Kanowski M. Tempelmann C. (2004). Eddy current correction in diffusion-weighted imaging using pairs of images acquired with opposite diffusion gradient polarity. *Magn. Reson. Med.* 51 188–193. 10.1002/mrm.10690 14705060

[B16] Calhoun V. D. Wager T. D. Krishnan A. Rosch K. S. Seymour K. E. Beth Nebel M. (2017). The impact of T1 versus EPI spatial normalization templates for fMRI data analyses. *Hum. Brain Mapp.* 38 5331–5342. 10.1002/hbm.23737 28745021 PMC5565844

[B17] Caruyer E. Lenglet C. Sapiro G. Deriche R. (2013). Design of multishell sampling schemes with uniform coverage in diffusion MRI. *Magn. Reson. Med.* 69 1534–1540. 10.1002/mrm.24736 23625329 PMC5381389

[B18] Catani M. Thiebaut de Schotten M. (2008). A diffusion tensor imaging tractography atlas for virtual in vivo dissections. *Cortex* 44 1105–1132. 10.1016/j.cortex.2008.05.004 18619589

[B19] Chang L. C. Jones D. K. Pierpaoli C. (2005). RESTORE: Robust estimation of tensors by outlier rejection. *Magn. Reson. Med.* 53 1088–1095. 10.1002/mrm.20426 15844157

[B20] Christiaens D. Reisert M. Dhollander T. Sunaert S. Suetens P. Maes F. (2015). Global tractography of multi-shell diffusion-weighted imaging data using a multi-tissue model. *Neuroimage* 123 89–101. 10.1016/j.neuroimage.2015.08.008 26272729

[B21] Cusack R. Brett M. Osswald K. (2003). An evaluation of the use of magnetic field maps to undistort echo-planar images. *Neuroimage* 18 127–142. 10.1006/nimg.2002.1281 12507450

[B22] Daducci A. Palù A. D. Lemkaddem A. Thiran J. Member S. (2015). COMMIT: convex optimization modeling for microstructure informed tractography. *IEEE Trans. Med. Imaging* 34 246–257. 10.1109/TMI.2014.2352414 25167548

[B23] Embleton K. V. Haroon H. A. Morris D. M. Ralph M. A. L. Parker G. J. M. (2010). Distortion correction for diffusion-weighted MRI tractography and fMRI in the temporal lobes. *Hum. Brain Mapp.* 31 1570–1587. 10.1002/hbm.20959 20143387 PMC6870737

[B24] Farzinfar M. Oguz I. Smith R. G. Verde A. R. Dietrich G. Gupta A. (2013). Diffusion imaging quality control via entropy of principal direction distribution. *Neuroimage* 82 1–12. 10.1016/j.neuroimage.2013.05.022 23684874 PMC3798052

[B25] Fillard P. Descoteaux M. Goh A. Gouttard S. Jeurissen B. Malcolm J. (2011). Quantitative evaluation of 10 tractography algorithms on a realistic diffusion MR phantom. *Neuroimage* 56 220–234. 10.1016/j.neuroimage.2011.01.032 21256221

[B26] Fischer C. Operto G. Laguitton S. Perrot M. Denghien I. Riviere D. (2012). “Morphologist 2012: the new morphological pipeline of BrainVISA,” in *Proceedings of the HBM*, Beijing.

[B27] Garyfallidis E. Brett M. Amirbekian B. Rokem A. van der Walt S. Descoteaux M. (2014). Dipy, a library for the analysis of diffusion MRI data. *Front. Neuroinformatics* 8:8. 10.3389/fninf.2014.00008 24600385 PMC3931231

[B28] Geffroy D. Rivière D. Denghien I. Souedet N. Laguitton S. Cointepas Y. (2011). “BrainVISA?: a complete software platform for neuroimaging,” in *Proceedings of the Python in Neuroscience Workshop*, Paris.

[B29] Ghosh A. Deriche R. (2016). A survey of current trends in diffusion MRI for structural brain connectivity. *J. Neural Eng.* 13:011001. 10.1088/1741-2560/13/1/011001 26695367

[B30] Girard G. Daducci A. Petit L. Thiran J. Whittingstall K. Deriche R. (2017). AxTract?: towards microstructure informed tractography. *Hum. Brain Mapp.* 38 5485–5500. 10.1002/hbm.23741 28766853 PMC6866984

[B31] Girard G. Whittingstall K. Deriche R. Descoteaux M. (2014). Towards quantitative connectivity analysis: reducing tractography biases. *Neuroimage* 98 266–278. 10.1016/j.neuroimage.2014.04.074 24816531

[B32] Glasser M. F. Smith S. M. Marcus D. S. Andersson J. Auerbach E. Behrens T. (2016). The Human Connectome Project’s neuroimaging approach. *Nat. Neurosci.* 19 1175–1187. 10.1038/nn.4361 27571196 PMC6172654

[B33] Graham M. S. Drobnjak I. Zhang H. (2016). Realistic simulation of artefacts in diffusion MRI for validating post-processing correction techniques. *Neuroimage* 125 1079–1094. 10.1016/j.neuroimage.2015.11.006 26549300

[B34] Hagmann P. Cammoun L. Gigandet X. Gerhardb S. Grant P. Wedeend V. (2010). MR connectomics: principles and challenges. *J. Neurosci. Methods* 194 34–45. 10.1016/j.jneumeth.2010.01.014 20096730

[B35] Haselgrove J. C. Moore J. R. (1996). Correction for distortion of echo-planar images used to calculate the apparent diffusion coefficient. *Magn. Reson. Med.* 36 960–964. 10.1002/mrm.1910360620 8946363

[B36] Hutchinson E. B. Avram A. V. Irfanoglu M. O. Koay C. G. Barnett A. S. Komlosh M. E. (2017). Analysis of the effects of noise, DWI sampling, and value of assumed parameters in diffusion MRI models. *Magn. Reson. Med.* 78 1767–1780. 10.1002/mrm.26575 28090658 PMC6084345

[B37] Irfanoglu M. O. Walker L. Sarlls J. Marenco S. Pierpaoli C. (2012). Effects of image distortions originating from susceptibility variations and concomitant fields on diffusion MRI tractography results. *Neuroimage* 61 275–288. 10.1016/j.neuroimage.2012.02.054 22401760 PMC3653420

[B38] Jenkinson M. Beckmann C. F. Behrens T. E. J. Woolrich M. W. Smith S. M. (2012). FSL. *Neuroimage* 62 782–790. 10.1016/j.neuroimage.2011.09.015 21979382

[B39] Jezzard P. Balaban R. S. (1995). Correction for geometric distortion in echo-planar images from B*0* Field Variations. *Magn. Reson. Med.* 34 65–73. 10.1002/mrm.1910340111 7674900

[B40] Jezzard P. Barnett A. S. Pierpaoli C. (1998). Characterization of and correction for eddy current artifacts in echo planar diffusion imaging. *Magn. Reson. Med.* 39 801–812. 10.1002/mrm.1910390518 9581612

[B41] Jones D. K. Cercignani M. (2010). Twenty-five pitfalls in the analysis of diffusion MRI data. *NMR Biomed.* 23 803–820. 10.1002/nbm.1543 20886566

[B42] Kim D.-J. Park H. Kang K. Shin Y. Kim J. Moon W. (2006). How does distortion correction correlate with anisotropic indices? A diffusion tensor imaging study. *Magn. Reson. Imaging* 24 1369–1376. 10.1016/j.mri.2006.07.014 17145409

[B43] Kybic J. Thévenaz P. Nirkko A. Unser M. (2000). Unwarping of unidirectionally distorted EPI images. *IEEE Trans. Med. Imaging* 19 80–93. 10.1109/42.836368 10784280

[B44] Lazar M. Alexander A. L. (2005). Bootstrap white matter tractography (BOOT-TRAC). *Neuroimage* 24 524–532. 10.1016/j.neuroimage.2004.08.050 15627594

[B45] Le Bihan D. Poupon C. Amadon A. Lethimonnier F. (2006). Artifacts and pitfalls in diffusion MRI. *J. Magn. Reson. Imaging* 24 478–488. 10.1002/jmri.20683 16897692

[B46] Leemans A. Jones D. K. (2009). The B -matrix must be rotated when correcting for subject motion in DTI data. *Magn. Reson. Med.* 61 1336–1349. 10.1002/mrm.21890 19319973

[B47] Mahmoudzadeh A. P. Kashou N. H. (2013). Evaluation of interpolation effects on upsampling and accuracy of cost functions-based optimized automatic image registration. *Int. J. Biomed. Imaging* 2013:395915. 10.1155/2013/395915 24000283 PMC3747392

[B48] Mangin J.-F. Fillard P. Cointepas Y. Le Bihan D. Frouin V. Poupon C. (2013). Toward global tractography. *Neuroimage* 80 290–296. 10.1016/j.neuroimage.2013.04.009 23587688

[B49] Manjón J. V. Coupé P. Concha L. Buades A. Collins D. L. Robles M. (2013). Diffusion weighted image denoising using overcomplete local PCA. *PLoS One* 8:e73021. 10.1371/journal.pone.0073021 24019889 PMC3760829

[B50] Mattes D. Haynor D. R. Vesselle H. Lewellen T. K. Eubank W. (2001). “Nonrigid multimodality image registration,” in *Proceedings of the SPIE International Society for Optical Engineering* Vol. 4322 (San Diego, CA), 1609–1620.

[B51] Merhof D. Soza G. Stadlbauer A. Greiner G. Nimsky C. (2007). Correction of susceptibility artifacts in diffusion tensor data using non-linear registration. *Med. Image Anal.* 11 588–603. 10.1016/j.media.2007.05.004 17664081

[B52] Modat M. Ridgway G. R. Taylor Z. A. Lehmann M. Barnes J. Hawkes D. (2010). Fast free-form deformation using graphics processing units. *Comput. Methods Programs Biomed.* 98 278–284. 10.1016/j.cmpb.2009.09.002 19818524

[B53] Mori S. Wakana S. van Zijl C. M. Nagae-Poetscher L. M. (2005). *MRI Atlas of Human White Matter.* Amsterdam: Elsevier.

[B54] Neher P. F. Descoteaux M. Houde J. Stieltjes B. Maier-hein K. H. (2015). Strengths and weaknesses of state of the art fiber tractography pipelines – A comprehensive in-vivo and phantom evaluation study using Tractometer. *Med. Image Anal.* 26 287–305. 10.1016/j.media.2015.10.011 26599155

[B55] Nilsson M. Szczepankiewicz F. Van Westen D. Hansson O. (2015). Extrapolation-based references improve motion and eddy-current correction of high B-value DWI data: application in Parkinson’s disease dementia. *PLoS One* 10:e0141825. 10.1371/journal.pone.0141825 26528541 PMC4631453

[B56] Ning L. Laun F. Gur Y. DiBella E. Deslauriers-Gauthier S. Megherbi T. (2015). Sparse Reconstruction Challenge for diffusion MRI: Validation on a physical phantom to determine which acquisition scheme and analysis method to use? *Med. Image Anal.* 26 316–331. 10.1016/j.media.2015.10.012 26606457 PMC4679726

[B57] Oguz I. Farzinfar M. Matsui J. Budi F. Liu Z. Gerig G. (2014). DTIPrep: quality control of diffusion-weighted images. *Front. Neuroinformatics* 8:4. 10.3389/fninf.2014.00004 24523693 PMC3906573

[B58] Papadakis N. G. Martin K. M. Wilkinson I. D. Huang C. L.-H. (2003). A measure of curve fitting error for noise filtering diffusion tensor MRI data. *J. Magn. Reson.* 164 1–9. 10.1016/S1090-7807(03)00202-7 12932449

[B59] Reber P. J. Wong E. C. Buxton R. B. Frank L. R. (1998). Correction of off resonance-related distortion in echo-planar imaging using EPI-based field maps. *Magn. Reson. Med.* 39 328–330. 10.1002/mrm.1910390223 9469719

[B60] Reisert M. Mader I. Anastasopoulos C. Weigel M. Schnell S. Kiselev V. (2011). Global fiber reconstruction becomes practical. *Neuroimage* 54 955–962. 10.1016/j.neuroimage.2010.09.016 20854913

[B61] Rivière D. Geffroy D. Denghien I. Souedet N. Cointepas Y. (2011). “Anatomist: a python framework for interactive 3D visualization of neuroimaging data,” in *Proceedings of the Python in Neuroscience Workshop*, Paris.

[B62] Roalf D. R. Quarmley M. Elliott M. A. Satterthwaite T. D. Vandekar S. N. Ruparel K. (2016). The impact of quality assurance assessment on diffusion tensor imaging outcomes in a large-scale population-based cohort. *Neuroimage* 125 903–919. 10.1016/j.neuroimage.2015.10.068 26520775 PMC4753778

[B63] Rohde G. K. Barnett A. S. Basser P. J. Marenco S. Pierpaoli C. (2004). Comprehensive approach for correction of motion and distortion in diffusion-weighted MRI. *Magn. Reson. Med.* 51 103–114. 10.1002/mrm.10677 14705050

[B64] Schmitt F. Stehling M. K. Turner R. (1998). *Echo-Planar Imaging.* Berlin: Springer.

[B65] Shen Y. Larkman D. J. Counsell S. Pu I. M. Edwards D. Hajnal J. V. (2004). Correction of high-order eddy current induced geometric distortion in diffusion-weighted echo-planar images. *Magn. Reson. Med.* 52 1184–1189. 10.1002/mrm.20267 15508159

[B66] Smith S. M. Jenkinson M. Woolrich M. W. Beckmann C. F. Behrens T. E. J. Johansen-Berg H. (2004). Advances in functional and structural MR image analysis and implementation as FSL. *Neuroimage* 23 S208–S219. 10.1016/j.neuroimage.2004.07.051 15501092

[B67] Sotiropoulos S. N. Jbabdi S. Xu J. Andersson J. Moeller S. Auerbach E. (2013). Advances in diffusion MRI acquisition and processing in the Human Connectome Project. *Neuroimage* 80 125–143. 10.1016/j.neuroimage.2013.05.057 23702418 PMC3720790

[B68] Tao R. Fletcher P. T. Gerber S. Whitaker R. T. (2009). “A Variational image-based approach to the correction of susceptibility artifacts in the alignment of diffusion weighted and structural MRI,” in *Information Processing in Medical Imaging. IPMI 2009. Lecture Notes in Computer Science* Vol. 5636 eds Prince J. L. Pham D. L. Myers K. J. (Berlin: Springer), 664–675. 10.1007/978-3-642-02498-6_55 PMC4031680 19694302

[B69] Taylor P. A. Alhamud A. van der Kouwe A. Saleh M. G. Laughton B. Meintjes E. (2016). Assessing the performance of different DTI motion correction strategies in the presence of EPI distortion correction. *Hum. Brain Mapp.* 37 4405–4424. 10.1002/hbm.23318 27436169 PMC5118068

[B70] Treiber J. M. White N. S. Steed T. C. Bartsch H. Holland D. Farid N. (2016). Characterization and correction of geometric distortions in 814 Diffusion Weighted Images. *PLoS One* 11: e0152472. 10.1371/journal.pone.0152472 27027775 PMC4814112

[B71] Van Essen D. C. Smith S. M. Barch D. M. Behrens T. E. J. Yacoub E. Ugurbil K. (2013). The WU-Minn human connectome project: an overview. *Neuroimage* 80 62–79. 10.1016/j.neuroimage.2013.05.041 23684880 PMC3724347

[B72] Wang S. Peterson D. J. Gatenby J. C. Li W. Grabowski T. J. Madhyastha T. M. (2017). Evaluation of Field Map and Nonlinear Registration Methods for Correction of Susceptibility Artifacts in Diffusion MRI. *Front. Neuroinformatics* 11:17. 10.3389/fninf.2017.00017 28270762 PMC5318394

[B73] Wu M. Chang L. C. Walker L. (2008). Comparison of EPI distortion correction methods in diffusion tensor MRI using a novel framework. *Med. Image Comput. Comput. Assist. Interv.* 11 321–329. 10.1007/978-3-540-85990-1-39 18982621 PMC4819327

[B74] Yamada H. Abe O. Shizukuishi T. Kikuta J. Shinozaki T. (2014). Efficacy of distortion correction on diffusion imaging?: comparison of FSL Eddy and Eddy_Correct Using 30 and 60 directions diffusion encoding. *PLoS One* 9:e112411. 10.1371/journal.pone.0112411 25405472 PMC4236106

[B75] Yendiki A. Koldewyn K. Kakunoori S. Kanwisher N. Fischl B. (2014). Spurious group differences due to head motion in a diffusion MRI study. *Neuroimage* 88 79–90. 10.1016/j.neuroimage.2013.11.027 24269273 PMC4029882

