# Supplementary material for: Diffusion MRI: Assessment of the Impact of Acquisition and Preprocessing Methods Using the BrainVISA-Diffuse Toolbox
Source: Front Neurosci. 2019 Jun 7;13:536. doi: 10.3389/fnins.2019.00536 (PMC6593278; doi:10.3389/fnins.2019.00536)
Supplement: Supplementary file 1 [file Data_Sheet_1.docx]

Supplementary material

# **S1.Clinical data acquisition settings**

# The MRI images of a healthy volunteer were acquired using the same sequences as in the HCP dataset with clinical settings. The subject was scanned in a Siemens Prisma 3T scanner with a maximum gradient strength of 80 mT/m, slew rate of 200 T/m/s and a 64-channel head coil. T1-weighted images were acquired using sagittal acquisition (TR/TI/TE = 2400/1040/2,34ms, flip angle = 8°, FO = 256x256 mm², 0.8mm in-plane resolution, slice thickness: 0.7mm, matrix: 320x320). The T1w images were resliced to a resolution of 1x1x2 mm for the experiments. Diffusion-weighted images were acquired with a spin-echo EPI sequence consisting of 3 shells of 60 diffusion-weighted volumes each (b=1000, 2000 and 3000 s/mm2) and 6 interleaved b0 volumes (TR/TE = 2610/73.6 ms, resolution: 2mm isotropic, FOV = 210x210mm2, matrix: 110x110, 66 axial slices, multiband factor = 2, partial Fourier = 6/8, echo spacing = 0.56ms). Gradients directions were sampled over the entire sphere, using the electrostatic repulsion method [27]. The entire diffusion sequence was repeated twice with RPE (A->P, P->A). A B0 field map image was also acquired using a dual-echo gradient-echo sequence (with delta TE = 2.46ms, resolution: 2 mm isotropic). As for the HCP data, 6 different subsets were extracted as detailed in Table 1 of the manuscript. In addition, we extracted 3 subsets, one for each b-value.

# **S2. Performance of distortion correction methods to recover brain geometry in clinical data**

**Figure S2.** **Average b0 image of the subject of the clinical dataset linearly (A) and non-linearly (B) registered into the structural space, after distortion correction through the six pipelines.** Grey-white interface (black line) and cortical surface (red/green line) of the non-distorted T1w image are overlaid on the b0 image. The following observations on data with A->P phase encoding are identical to the HCP data, which are in L->R phase encoding. (A) Susceptibility-induced distortions correction enables to recover the true geometry of the brain (red arrows). The signal intensity in stretched areas can be corrected using a B0 field map image (see empty arrows in the zoomed images). But only the use of a reversed phase-encoding acquisition (FSb0RPE and FSfullRPE) can properly reconstruct the signal in compressed areas (see full arrows). (B) Non-linear transformation is able to partially correct for residual geometric distortions in particular with a proper geometry of the frontal, temporal lobes and ventricles (green arrows).


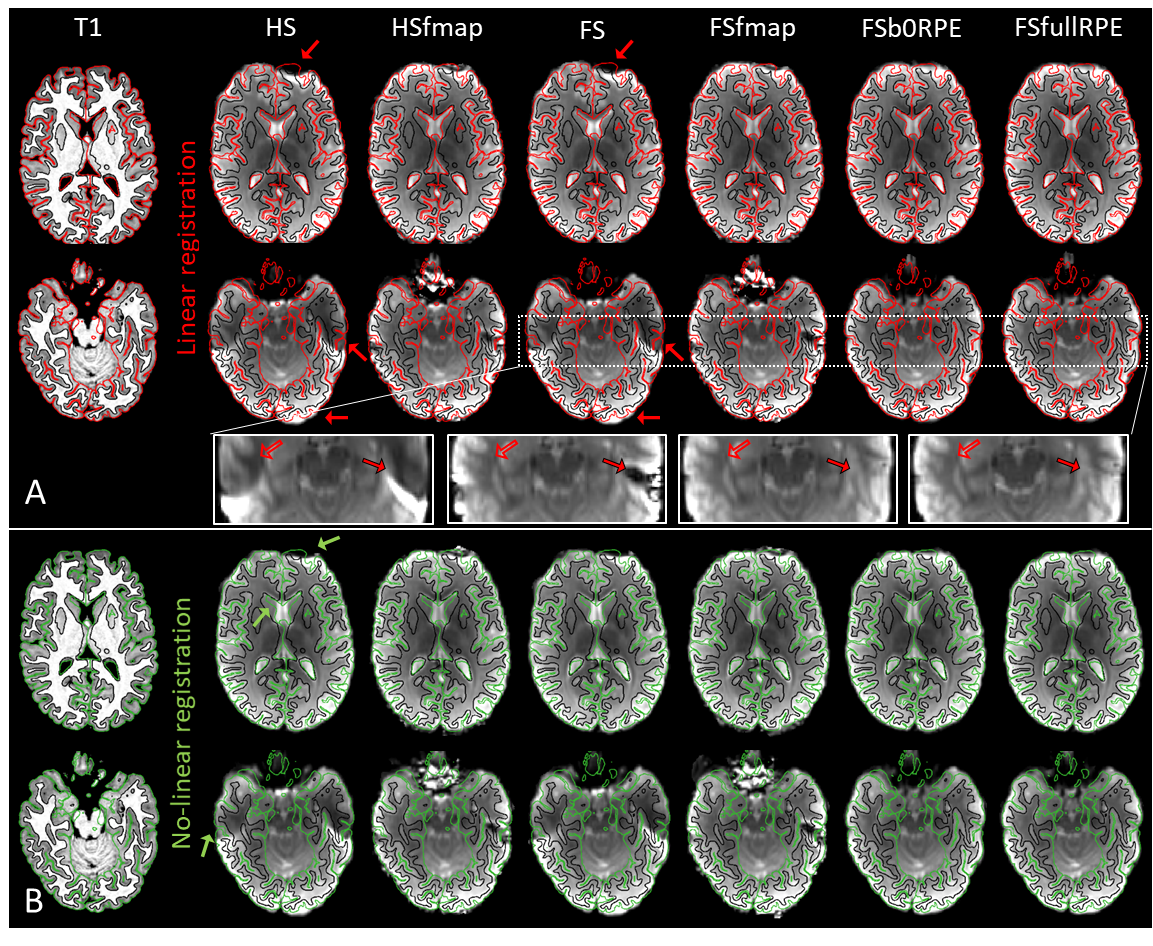


# **S3. Impact of head movement on co-registration quality**

As opposed to the preprocessed data which underwent motion correction, the uncorrected volumes are not aligned to each other. Thus, we only applied the registration into the structural space (estimated using the FA image) to the first b0 volume. This method induces that the SNR in the b0 image is lower than in the averaged b0 images used in the six pipelines. The Mattes Mutual Information (MMI) was then computed as a similarity metric between the b0 and the T1 image. Results with linear and non-linear registration are displayed in grey in Figure S1. They both show that only some subjects are impacted by the correction of movements and eddy-current-induced distortions. While the difference of SNR and the eddy-current distortions would have impacted all subjects, further examination of the movement parameters estimated during eddy-currents correction showed that these subjects also presented a more important cumulative displacement across volumes during the scan. This finding emphasizes the interest of motion correction, in particular here, for the registration quality with the structural space.

**Figure S3. Quantitative assessment of distortion correction methods using the Mattes Mutual Information. The similarity metric is computed between the T1w image and the (average) b0 image registered into the structural space, using affine transformation (left) or non-linear transformation (right). The results corresponding to uncorrected data are plotted in grey. The black line with red dots corresponds to the clinical dataset.**

**
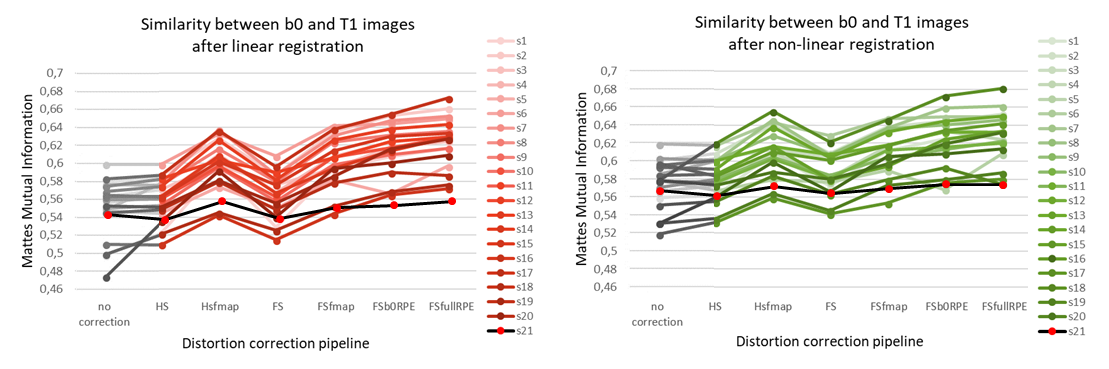
**

# **S4. Impact of head movement and eddy-current on diffusivity measurements**

As for the data preprocessed through the six pipelines, the weighted least-square tensor model was estimated on the uncorrected raw data. The averages of tensor-derived indices, namely the tensor fitting error (TFE), the mean dispersion index (MDI), the fractional anisotropy (FA) and the mean diffusivity (MD), were computed across all white matter voxels. Results are shown on Figure S2. As a first observation, we can see that the standard deviation of the values across subjects is importantly reduced for corrected data (91%, 46% and 64% reduction of std for respectively TFE, MDI, FA). As noticed in Figure S1, the four highly deviated subjects (for TFE and FA) still correspond to those who presented substantial movement during the acquisition. This finding suggests that TFE and FA are particularly sensitive to subject movement, while MDI and MD show less inter-subject variability. In accordance with (Kim et al., 2006; Rohde, Barnett, Basser, Marenco, & Pierpaoli, 2004), the TFE is significantly reduced for corrected compared to uncorrected data, which indicates that eddy-current distortions correction is mandatory to obtain a proper tensor model estimation. In line with (Yamada, Abe, Shizukuishi, Kikuta, & Shinozaki, 2014), we found an overall increase of FA values for corrected compared to uncorrected data.

**Figure S4. Effect of preprocessing methods on tensor-derived indices. For every subject represented with different colors, the mean values are plotted as a function of the preprocessing pipeline used to correct distortions. The results corresponding to uncorrected data are plotted in grey. The black line with red dots corresponds to the clinical dataset.**

**
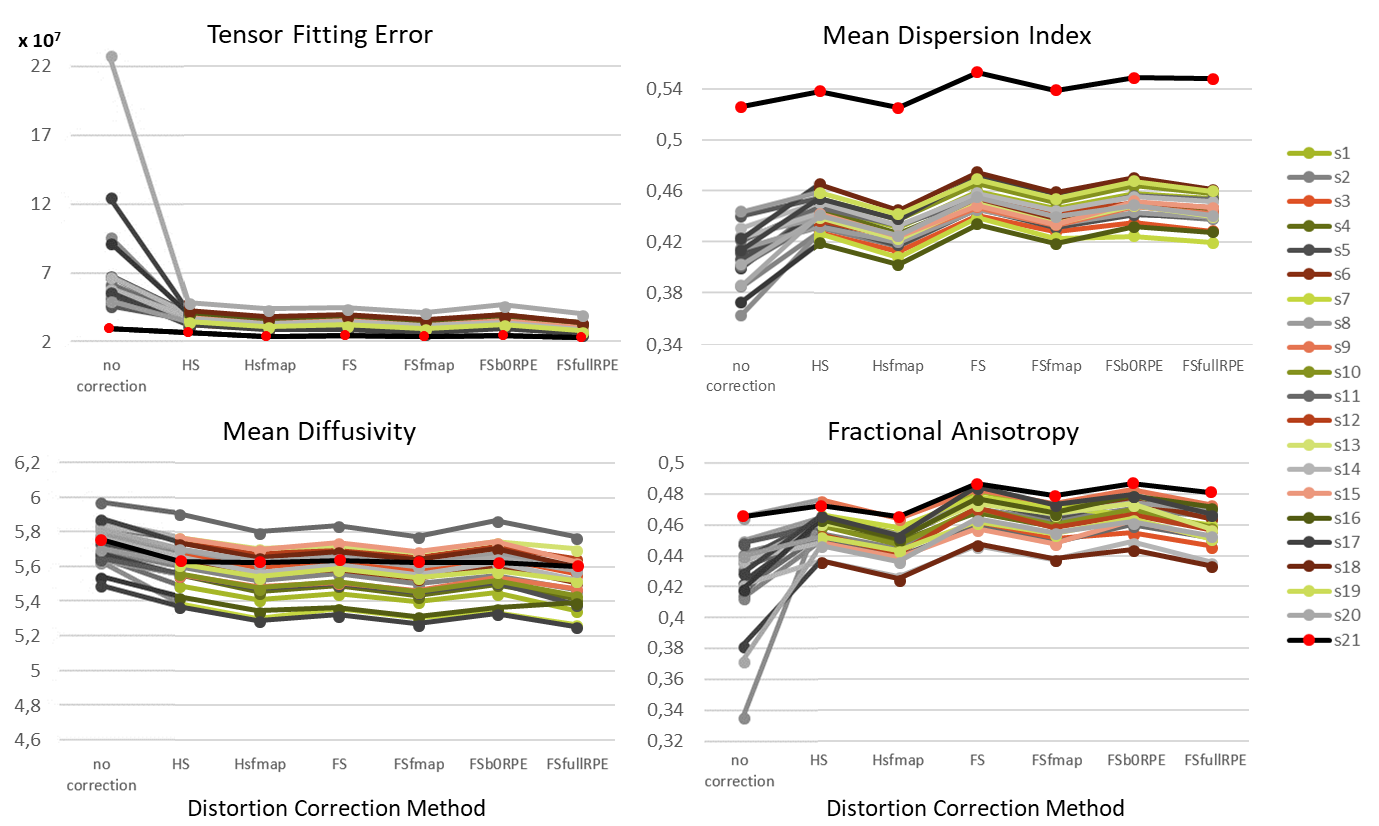
**

# **S5. Impact of spatial resolution on co-registration quality**

We divided the clinical dataset into three subsets for each of the b-values 1000, 2000 and 3000 s/mm². We performed the same analyses as for the full multi-shell subset. First, we found that the non-linear registration yielded an improved similarity between T1w and DW images. This finding emphasizes its interest even with a lower and non-isotropic spatial resolution of the T1w image. Second, and as expected, the b-value presented no impact on the similarity metric, which is computed using the b0 volume.

**Figure S5. Linear and non-linear registration between T1w and diffusion weighted images with low spatial resolutions and different b-values. The Mattes Mutual Information is used as similarity metric between the T1w image and the (average) b0 image registered into the structural space, using affine transformation (left) or non-linear transformation (right). The black line with red dots corresponds to the full clinical dataset. The results corresponding to each b-value are plotted in red.**

**
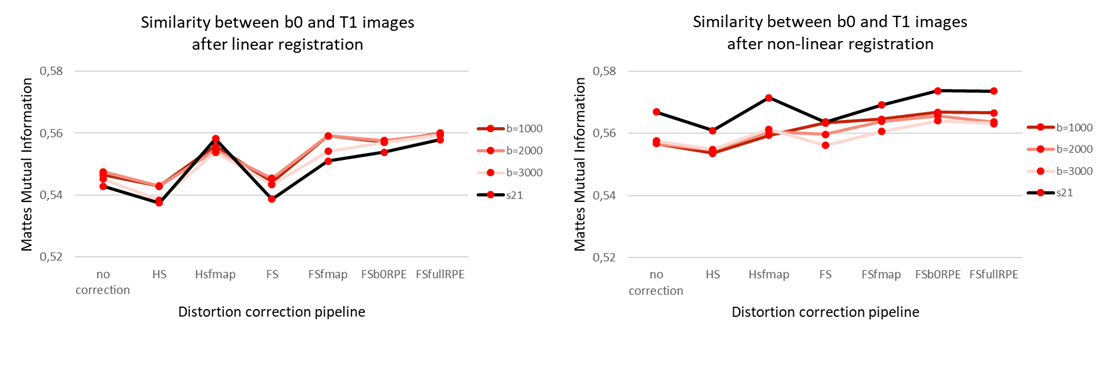
**

# **S6. Impact of b-value on diffusivity measurements**

As for the registration quality, we assessed the tensor-fitting quality for the multi-shell clinical dataset and for the three subsets of b-values 1000, 2000 and 3000 s/mm². First, we found a much lower TFE for the separated subsets compared to both the multi-shell dataset and the HCP data (note that the ordinate scale has been cut for clarity purpose). Also, the multi-shell dataset shows equivalent MD as the b=3000s/mm² subset and equivalent FA as the b=1000s/mm² subset. These findings demonstrate that mixing several b-values in the data, which is basically adapted to more complex diffusion models, has a large influence on the tensor model fitting. Second, we found that MD and FA values was largely dependent on the b-value, contrarily to the fitting performance metrics.

**Figure S6. Influence of b-value and spatial resolution of diffusion weighted images on tensor-derived indices. For the full dataset (black line with red dots) and for each b-value subset (light to dark red), the mean values are plotted as a function of the preprocessing pipeline used to correct distortions.**

**
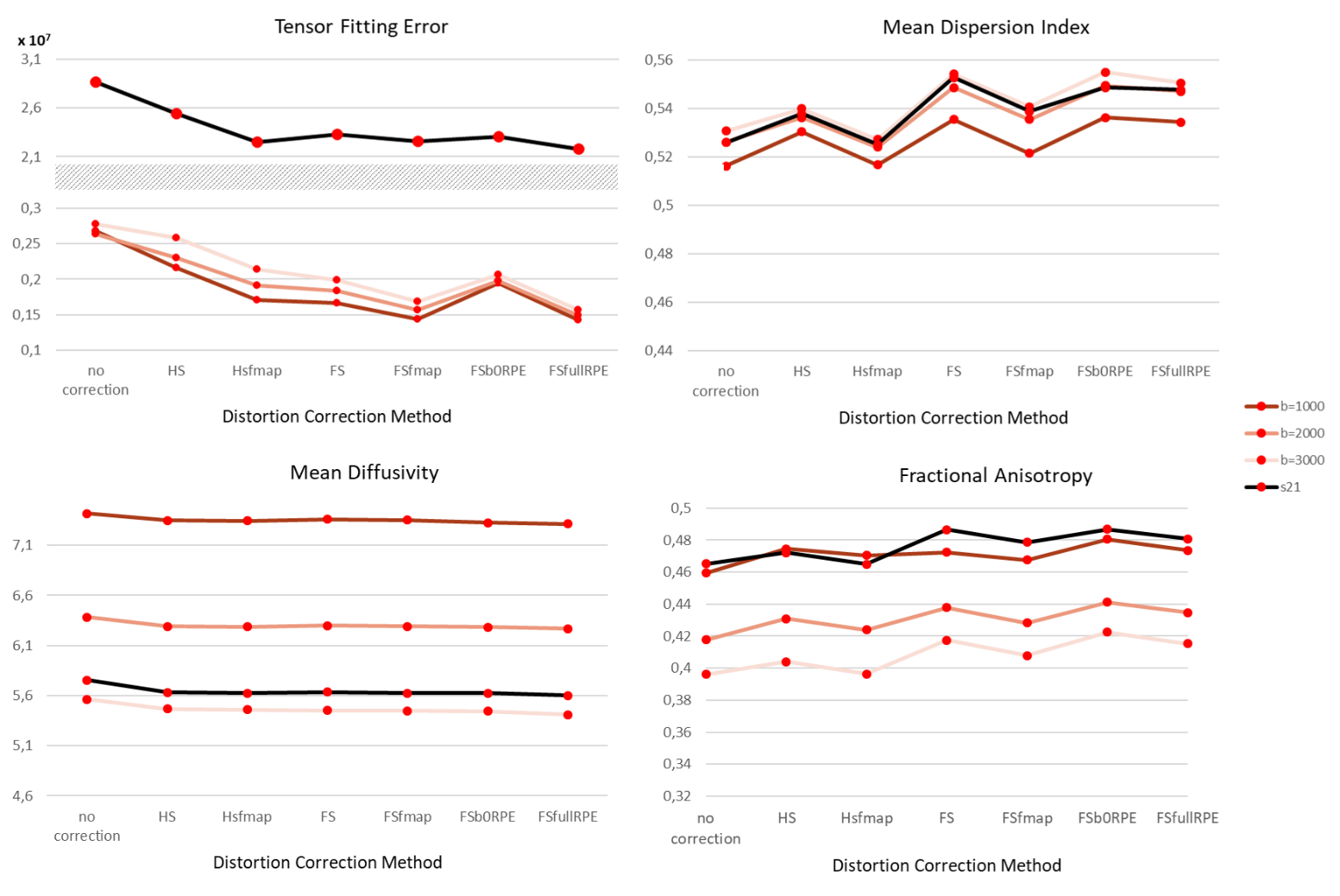
**

# **References**

Kim, D.-J., Park, H., Kang, K., Shin, Y., Kim, J.-J., Moon, W., … Kim, S. I. (2006). How does distortion correction correlate with anisotropic indices? A diffusion tensor imaging study. *Magnetic Resonance Imaging*, *24*(10), 1369–1376. https://doi.org/10.1016/j.mri.2006.07.014

Rohde, G. K., Barnett, A. S., Basser, P. J., Marenco, S., & Pierpaoli, C. (2004). Comprehensive approach for correction of motion and distortion in diffusion-weighted MRI. *Magnetic Resonance in Medicine*, *51*(1), 103–114. https://doi.org/10.1002/mrm.10677

Yamada, H., Abe, O., Shizukuishi, T., Kikuta, J., & Shinozaki, T. (2014). Efficacy of Distortion Correction on Diffusion Imaging : Comparison of FSL Eddy and Eddy _ Correct Using 30 and 60 Directions Diffusion Encoding, *9*(11). https://doi.org/10.1371/journal.pone.0112411
